# Increasing Crop Diversity Mitigates Weather Variations and Improves Yield Stability

**DOI:** 10.1371/journal.pone.0113261

**Published:** 2015-02-06

**Authors:** Amélie C. M. Gaudin, Tor N. Tolhurst, Alan P. Ker, Ken Janovicek, Cristina Tortora, Ralph C. Martin, William Deen

**Affiliations:** 1 Department of Plant Agriculture, University of Guelph, 50 Stone Road East, Guelph, ON, N1G2W1, Canada; 2 Department of Food, Agricultural and Resources Economics, University of Guelph, 50 Stone Road East, Guelph, ON, N1G2W1, Canada; 3 Department of Mathematics and Statistics, McMaster University, 1280 Main St W, Hamilton, ON, L8S4L8, Canada; Instituto de Agricultura Sostenible (CSIC), SPAIN

## Abstract

Cropping sequence diversification provides a systems approach to reduce yield variations and improve resilience to multiple environmental stresses. Yield advantages of more diverse crop rotations and their synergistic effects with reduced tillage are well documented, but few studies have quantified the impact of these management practices on yields and their stability when soil moisture is limiting or in excess. Using yield and weather data obtained from a 31-year long term rotation and tillage trial in Ontario, we tested whether crop rotation diversity is associated with greater yield stability when abnormal weather conditions occur. We used parametric and non-parametric approaches to quantify the impact of rotation diversity (monocrop, 2-crops, 3-crops without or with one or two legume cover crops) and tillage (conventional or reduced tillage) on yield probabilities and the benefits of crop diversity under different soil moisture and temperature scenarios. Although the magnitude of rotation benefits varied with crops, weather patterns and tillage, yield stability significantly increased when corn and soybean were integrated into more diverse rotations. Introducing small grains into short corn-soybean rotation was enough to provide substantial benefits on long-term soybean yields and their stability while the effects on corn were mostly associated with the temporal niche provided by small grains for underseeded red clover or alfalfa. Crop diversification strategies increased the probability of harnessing favorable growing conditions while decreasing the risk of crop failure. In hot and dry years, diversification of corn-soybean rotations and reduced tillage increased yield by 7% and 22% for corn and soybean respectively. Given the additional advantages associated with cropping system diversification, such a strategy provides a more comprehensive approach to lowering yield variability and improving the resilience of cropping systems to multiple environmental stresses. This could help to sustain future yield levels in challenging production environments.

## Introduction

Vulnerability of agroecosystems to variations in weather is of increasing concern as abnormal production scenarios associated with predicted changes in climate may become more common [1–8]. While northern corn and soybean production regions may benefit from a longer crop-growing period, additional summertime warming and heavy rainfall events may challenge productivity unless adaptive measures are taken. Projections from various climate models suggest that year-to-year variations will increase along with wetter spring conditions, drier summer months and greater frequency of abnormal precipitation events [9,10]. Across the Midwestern United States, the number of days with heavy rainfall more than tripled in the past 50 years, particularly in the spring [10]. Increases in summer temperatures are also projected to increase soil water evaporation and crop transpiration, further increasing soil water deficits and economic losses [11].

One key management strategy to potentially cope with impending climatic change is to increase agroecosystem diversity, particularly temporal diversity using more complex crop rotations. Greater diversity has been hypothesized to lead to larger stability of natural ecosystems [12–17] and similar benefits have been suggested in agroecosystem literature [18–21]. Agroecosystem stability refers to cropping systems ability to maintain yields after a stress period or perturbation and includes various concepts such as resilience, persistence and resistance [17]. Resilience is generally defined as the propensity of a system to retain its state following a perturbation [14]. Although the idea of building resilience has been studied in various natural ecosystems [13,22], communities and food systems [23–25], it has not been well studied at the field scale where stability and resilience are often used interchangeably to describe fluctuations in final crop yields after perturbance. As proposed in the literature, cropping systems are stable/resilient if they are able to retain yield potential and recover functional integrity (produce food and feed) when challenged by environmental stresses [18,26]. In our study, we designated as more stable, systems with lower variations or losses in final yield in response to specific weather pattern.

Winter wheat (*Triticum aestivum*) and spring cereals offer an opportunity to maintain diversity in corn and soybean–based cropping systems with potential to improve yield stability when environmental stresses occur [20]. Unfortunately, agroecosystem diversity has been decreasing throughout the Corn Belt, as illustrated in Iowa [21] and Ontario where small grains cereals have been increasingly replaced by corn and soybean in the last 30 years (S1 Fig., [27]). Although numerous long-term studies have described the effects of rotation complexity on corn and soybean yields [28–32], few have attempted to quantify the impact of those management practices on yield stability, especially when soil moisture is limiting or in excess. Inclusion of cereals grains and forage species into short corn-soybean rotations resulted in higher corn yields compared to continuous corn in dry years of several studies located in the northern Corn Belt [33–37]. However, these studies did not conclusively establish the effects of variation in weather patterns on yields and additional management effects were confounded. Other studies demonstrated the enhanced importance of rotation diversity on corn and soybean yields under dryland conditions [38–40]. However, the locations of these studies do not represent growing conditions in the northern Corn Belt where occurrence of excess soil moisture is more common.

Understanding the role of diversity on the functioning of agroecosystems and crop yield response to environmental stresses may help design cropping systems able to maintain provisioning and regulating ecosystem services under abnormal weather scenarios. The objectives of our study were to provide further insights on the linkages between diversity and stability by testing whether more diverse crop rotations are associated with greater yield stability over time and higher yields under abnormal weather conditions. We hypothesized that maintaining agroecosystem diversity provides productive responses to weather shocks and increase the probability of obtaining high corn yields while decreasing vulnerability of corn and soybean yields to weather variations. We used yield and weather data obtained from a 31-year long term rotation and tillage trial in Ontario (Elora) to measure the impact of temporal (rotation) and spacial (tillage) diversity on 1) downside risk and probabilities of corn yield advantage, 2) corn and soybean yields stability and 3) quantify potential rotation benefits at a northern Corn Belt location under different soil moisture and temperature scenarios.

## Material and Methods

### Long-term rotation and tillage experiment

**Fig 1:**
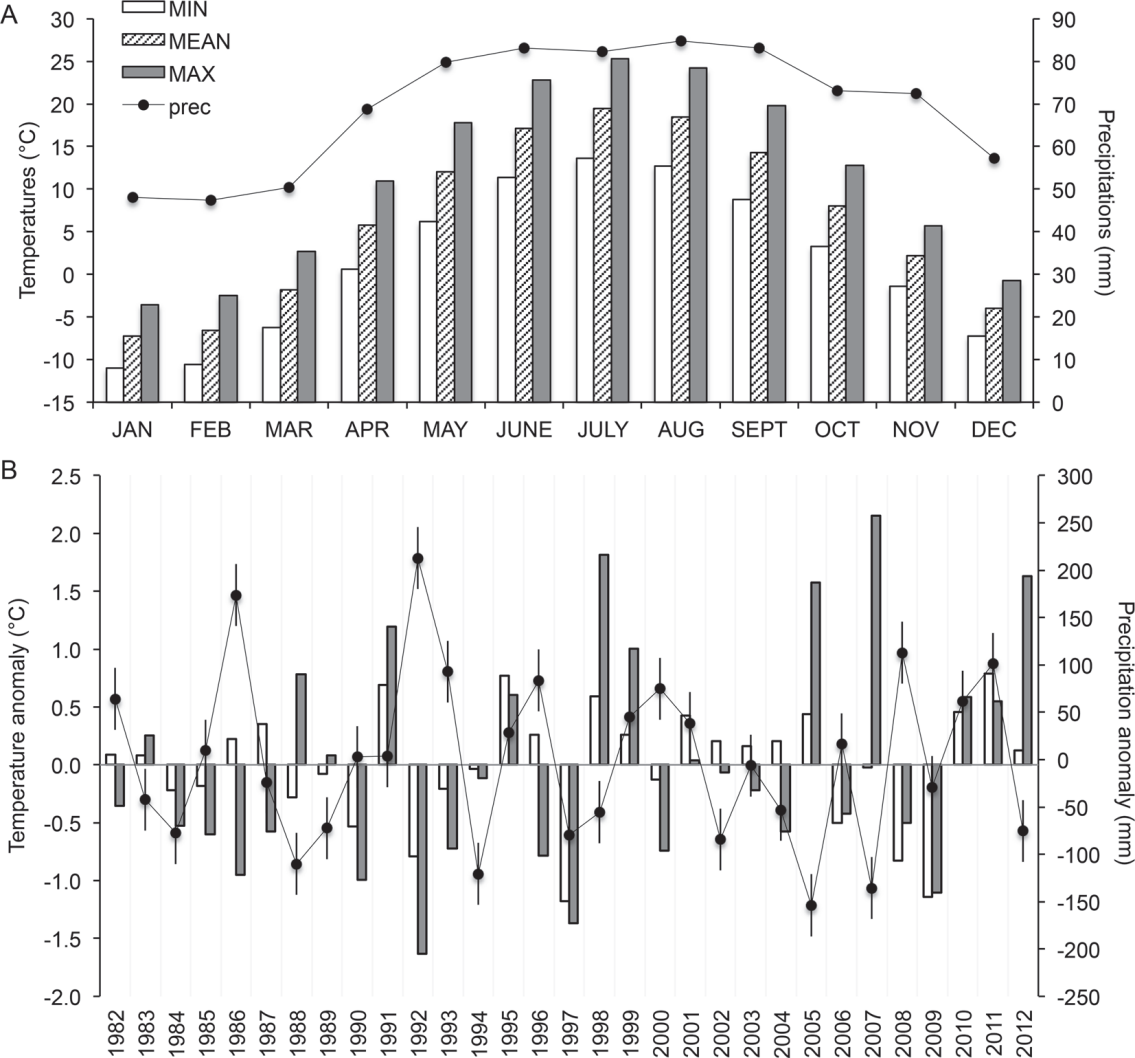
Temperature and total precipitation recorded at the experimental site from 1982 to 2012. (A) Monthly averaged 31-year temperature and precipitation and (B) anomalies during summer crop growing season. Deviation from 31-year average from May 1st to November 30th in minimum (MIN), maximum (MAX) and mean (MEAN) temperatures and total precipitation (prec) are shown. Error bars show Fisher protected LSD for total precipitation (32.7mm). Fisher protected LSD for maximum and minimum temperatures were 0.48°C and 0.20°C respectively.

Thirty one years (1982–2012) of crop yield response to two levels of tillage and seven different corn-based crop rotations were obtained from a long-term rotation and tillage trial located at the University of Guelph Elora Research Station located near Elora, ON, Canada (43°8'27.76 N, -80°24'20.43 W; elevation 376 m). The site was tile drained in 1967 and planted to corn continuously from 1967 to 1980. The soil is classified as Woolwich silt loam [28], Grey Brown Luvisol [41] or Albic Luvisol [42]. The trial was located in a temperate continental climate, characterized by homogeneously distributed precipitation during the growing season from April to November (102 mm on average) with cool springs and warm to hot summers (Fig. 1A).

The experiment was initiated in 1980 and designed as a randomized split-plot with four replications for each tillage–rotation combination. The first two years of the trial were considered as set up years and not included in the analysis. Seven rotation treatments, consisting of six 4-year rotations and continuous corn (CCCC), were randomly assigned to the main plots (16.7m x 6m). The cropping sequence in the 4-year rotations was comprised of 2 years of corn followed by one or two rotation crops with or without red clover cover crops (Table 1). Each of the 4-year rotations was duplicated 2 years out of phase so that corn and soybean yields were available for all rotations yearly and biyearly respectively.

**Table 1:** Long-term effects of rotation diversity and tillage on mean and cumulative corn and soybean yields.

		Cumulative yields (Mg ha^-1^)				Mean grain yields (kg ha^-1^)			
		Corn		Soybean		Corn		Soybean	
Rotation diversity		Till	Red. Till	Till	Red. Till	Till	Red. Till	Till	Red. Till
1 crop	CCCC	260.2 ^a^*	249.6 ^a^			8197.2 ^a^*	8052.6 ^a^		
2 crops	CCSS	263.5 ^a^*	256.4 ^a^	41.7 ^a^*	40.7 ^a^	8272.5 ^a^	8332.1 ^a^	2436.9 ^a^	2378.8 ^a^
	CCAA	276.8 ^b^	275.1 ^b^			8928.9 ^b^	8874.2 ^b^		
3 crops	CCSW	265.0 ^a^	265.1 ^ab^	44.3 ^b^	43.7 ^b^	8385.3 ^a^	8549.0 ^ab^	2754.2 ^b^	2719.7 ^b^
	CCOB	269.6 ^a^	265.6 ^ab^			8515.5 ^a^	8569.7 ^ab^		
3 crops, 1 cover crops	CCSWrc	275.9 ^b^	273.1 ^b^	45.1 ^b^	44.2 ^b^	8836.4 ^b^	8528.2 ^ab^	2791.7 ^b^	2734.6 ^b^
3 crops, 2 cover crops	CCOrcBrc	277.1 ^b^	274.2 ^b^			8899.4 ^b^	8811.7 ^b^		
Source of variation		df		df		df		df	
Year		30	***	30	**	30	***	30	***
Rotation		6	**	2	**	6	***	2	***
Tillage		1	**	1	**	1	**	1	***
Year x Rotation		180	**	60	**	180	***	60	**
Year x Tillage		30	**	30	**	30	***	30	***
Rotation x Tillage		6	**	2	**	6	**	2	**
Year x Rotation x Tillage		180	**	60	**	180	**	60	**

Shown are the Least Squared Means and cumulative yields (accumulated mean yields, 1982–2012) for corn (n = 31) and soybean (n = 16) obtained over a 31-year period (1982–2012). LS Means followed by the same letter are not significantly different at p = 0.05; (*) next to the mean indicate significant tillage effect. In ANOVA section, (**) significant effect at p<0.05 and (***) p<0.001. Crop abbreviations: C = Corn, S = Soybean, A = Alfalfa, W = Winter wheat, O = Oat, B = Spring barley, rc = under seeded red clover; *df* = degree of freedom, Till = continuous tillage, Red Till = reduced tillage (conservation tillage from 1982 to 2001 followed by no till from 2002 to 2012.

Each main rotation plot was split into two levels of tillage: conventional tillage and reduced tillage. Conservation tillage from 1980 to 2001 consisted of fall chisel plowing to a depth of 15–20 cm except before establishment of winter wheat where it consisted of 2 passes of a tandem disk to a depth of 10 cm. Starting in 2002 the conservation tillage system was converted to no-till. Conventional tillage consisted of moldboard plowing to a depth of 15–20 cm in the fall. Secondary tillage was done in both chisel and moldboard plow tillage systems and usually consisted of two passes of a field cultivator and packer within 1 day of crop seeding.

Plots were maintained so that fertility, pests and weed pressure did not differ between plots and that productivity was not adversely affected by those factors. For corn, 160 to 180 kg N ha^-1^, 23 to 32 kg P ha^-1^, and 50 to 90 kg K ha^-1^ were applied annually. Fertilizer inputs for the various rotation crops were applied at rates equal or higher than the recommended rates according to soil tests [43]. Crop varieties were changed to ensure that the trial represented farmer’s practices and the best available yield potential. The red clover cover crop was either frost seeded into winter wheat in late March or early April or drill-seeded simultaneously with oat or barley. For rotations with cereals underseeded with red clover, an additional fall application of glyphosate was applied to the reduced tillage subplots to minimize volunteer regrowth in the next year’s crop.

Grain moisture at harvest was determined using a GAC 2100 grain moisture tester (Dickey-John, Springfield, IL, USA) and final yields were adjusted to moisture content of 15.5% for corn, 13% for soybean, 14.5% for wheat and 14% for barley and oat.

### Modeling of environmental data

Total precipitation, average, maximum and minimum temperatures were recorded at the Elora Research Station since the onset of the experiment. Corn and soybean developmental stages were estimated based on crop heat units and planting, flowering and harvesting dates recorded over the past 30 years.

During the course of the experiment, averaged minimum and maximum temperatures during the growing seasons (April to November) were significantly below or above the 30-year average 20% and 33% of the time respectively (Fig. 1B). Total precipitation varied more than two fold and the average daily high and low temperatures up to 2°C (Fig. 1B).

Average soil moisture deficit for each growing season was modeled using the Van Genuchten [44] and Mualem [45] hydraulic functions built in the Hydrus 1D modeling environment (PC Progress, Prague, Czech Republic, S2 Fig.). Precipitation water flow in the soil matrix was analyzed assuming a 100 cm soil zone, a maximum root depth of 100cm for corn and 50cm for soybean and surface runoff and free drainage as upper and bottom boundary respectively. The rainfall interception was set at 0.75mm with a constant Albedo of 0.23. The Feddes and Zaradny [46] model with Wesseling [47] parameters was used to simulate root and water uptake while soil cover fractions were calculated using corn and soybean specific crop coefficients. Field capacity and permanent wilting points were used to determine the volumetric soil water content at 25% available soil water (θ = 0.21 or 21%) which has been shown to be the most accurate threshold to categorize water stress effects on corn yield across environments in Ontario [48]. Estimated volumetric soil moisture available for corn progressively decreased below 25% of Available Soil Water (ASW) during five growing seasons, especially in 2012 where corn remained below this threshold for 63 days after silking (S2 Fig.). Estimated volumetric water content remained between 50%-25% ASW thresholds under soybean with similar years being the driest (S2 Fig.).

### Statistical procedures

Hierarchical clustering and PCA

Hierarchical clustering algorithms were used to identify the different weather patterns that occurred over the experimental period (S3, S4 Figs.) using Matlab R2012a. Years were clustered using normalized soil moisture estimated by the Hydrus 1 model and normalized average temperature during distinct corn and soybean developmental stages. Environmental data was normalized using the following formula:

$$\tilde{X}=\frac{X-\min\left(X_{i}\right)}{\max\left(X_{i}\right)-\min\left(X_{i}\right)}$$

Euclidian distances and Ward linkage functions were use to produce nested clustering structures [49]. Ward's method minimizes the increase in the total within-cluster error sum of squares, creating clusters with the lowest variance. Cluster partition was achieved using the best cut method based on dendograms of the aggregative structure [49] (S3, S4 Figs.). Five clusters for corn and six clusters for soybean significantly captured growing season variations in temperature and soil moisture during crop growth (S3, S4 Figs.).

Principal component analysis (R Agricolae package version 1.2–1) [50]) was performed to describe the variability of the year data matrix (S2 Fig.) [51]. Maximum temperatures during flowering significantly explained 61% and 46% of the variation in soil moisture available to corn and soybean respectively with significant carryover effects later in the season (S2 Fig.).

ANOVA and yield stability

Analysis of variance was performed using R Agricolae package (version 1.2–1) [50]. Crop yields were analyzed using repeated-measurements analysis of variance using Mixed model with rotation and tillage as fixed effects and year as random effect. Prior to the analysis of variance, residuals were found homogeneous and normally distributed based on the Shapiro–Wilk test (p = 0.925) and no significant outliers were detected by Lund’s test. Least Square means (LS mean) and cumulative yields (accumulated LS mean yield from 1982 to 2012) were generated. Yield variation (CV) under specific weather patterns represented by year clusters was calculated for each rotation and tillage treatment$CV\%=100\left(\sigma n^{-1/2}\right)$. Rotation benefits were defined as corn and soybean Δ yield compared to CCSS rotation and expressed as % of CCSS yields for each cluster. Differences between clusters and treatments were tested using the Tukey HSD test. Type I error for all statistical tests was set at p = 0.05.

Probability of high and low corn yield events

Statistical analyses were performed with custom made scripts in R (version 3.1.1, S1 Code). Probability densities of corn yields from the trial were estimated using a finite mixture model of normal distributions with two components:

$$y_{t}∼\lambda N\left(\mu_{1},\sigma_{1}^{2}\right)+\left(1-\lambda \right)N\left(\mu_{2},\sigma_{2}^{2}\right)$$

where $y_{t}$ is a series of yield observations over time, λ is a weighting parameter, and $N\left(∙\right)$ is the Normal distribution for each cluster parameterized with its own component mean, $\mu_{j}$, and variance, $\sigma_{j}^{2}$. Each component of the mixture model corresponds to a subpopulation within the sample data.

The choice of two components was based on three empirical tests [52] (S1 Table). Unknown parameters were estimated with a maximum likelihood approach using the Expectations-Maximization (EM) algorithm [53], with estimated parameters for each individual treatment (14 treatments with n = 31) and pooled across treatments (n = 434) are reported in S2 Table. Four different initialization methods were used for the algorithm and parameter estimates were based on the set of starting values giving the highest penalized likelihood [46]. Yield distributions were estimated using yields adjusted for a time trend without treatment effects (because the treatment fixed effects failed to provide a statistically significant improvement in successive F-tests, S3 Table). The qualitative conclusions of the analysis were consistent under a number of different trend and density estimation approaches (S4–S7 Tables).

To compare performance of different treatments at different points on the support of the yield distribution, three score metrics were constructed: 1) probability of high yield, P(hy), was defined as the estimated probability of achieving yields above the 90^th^ percentile of the pooled data (11321.4 kg ha^-1^); 2) probability of “downside potential”, P(DP), as the estimated probability yields from a given treatment fall into the lower component of the pooled yield distribution estimate; 3) probability of high yield, P(ly), was defined as the estimated probability of achieving yields below the 10^th^ percentile of the pooled data (7546.7 kg ha^-1^). These metrics are all based on the estimated yield probability densities using detrended data. To capture multidimensional probabilities into one value, performance indexes (Pi) were calculated based on ordinal ranks for each metric on a 100-point scale as follows:

$$\mathrm{Pi}=104-\left(2\left(\text{Rank}\;P{(}_{\mathrm{HY}})\right)+\text{Rank}\;P{(}_{\mathrm{DP}})+\text{Rank}\;P{(}_{\mathrm{LY}})\right)$$

An indication of each metric’s statistical significance is provided using *p*-values generated with randomization methods [54]. The p-values were constructed with 5,000 randomized yield sets for which parameters, densities, and metrics were estimated. The p-values represent the per cent of randomized metrics higher or lower than the treatment metrics.

The analysis was conducted for corn only, since the sample for soybean (n = 16 per treatment) was not sufficiently large.

## Results

### Crop diversity increases corn and soybean yields over time

Rotation, tillage, year and their interactions had a significant effect on cumulative and mean yearly corn and soybean yields (Table 1). Compared to CCSS, representative in our trial of the most common crop rotation in Ontario (CS), rotations with the highest degree of complexity or containing alfalfa had significantly higher cumulative and mean corn and soybean yields, especially when tillage was applied (Table 1). Addition of forage legumes into the tilled system significantly increased cumulative and mean corn yields by 4% and 6%, respectively, compared to other rotations. Diversification of a corn-soybean rotation with wheat increased mean soybean yields by 13% (Table 1).

**Fig 2:**
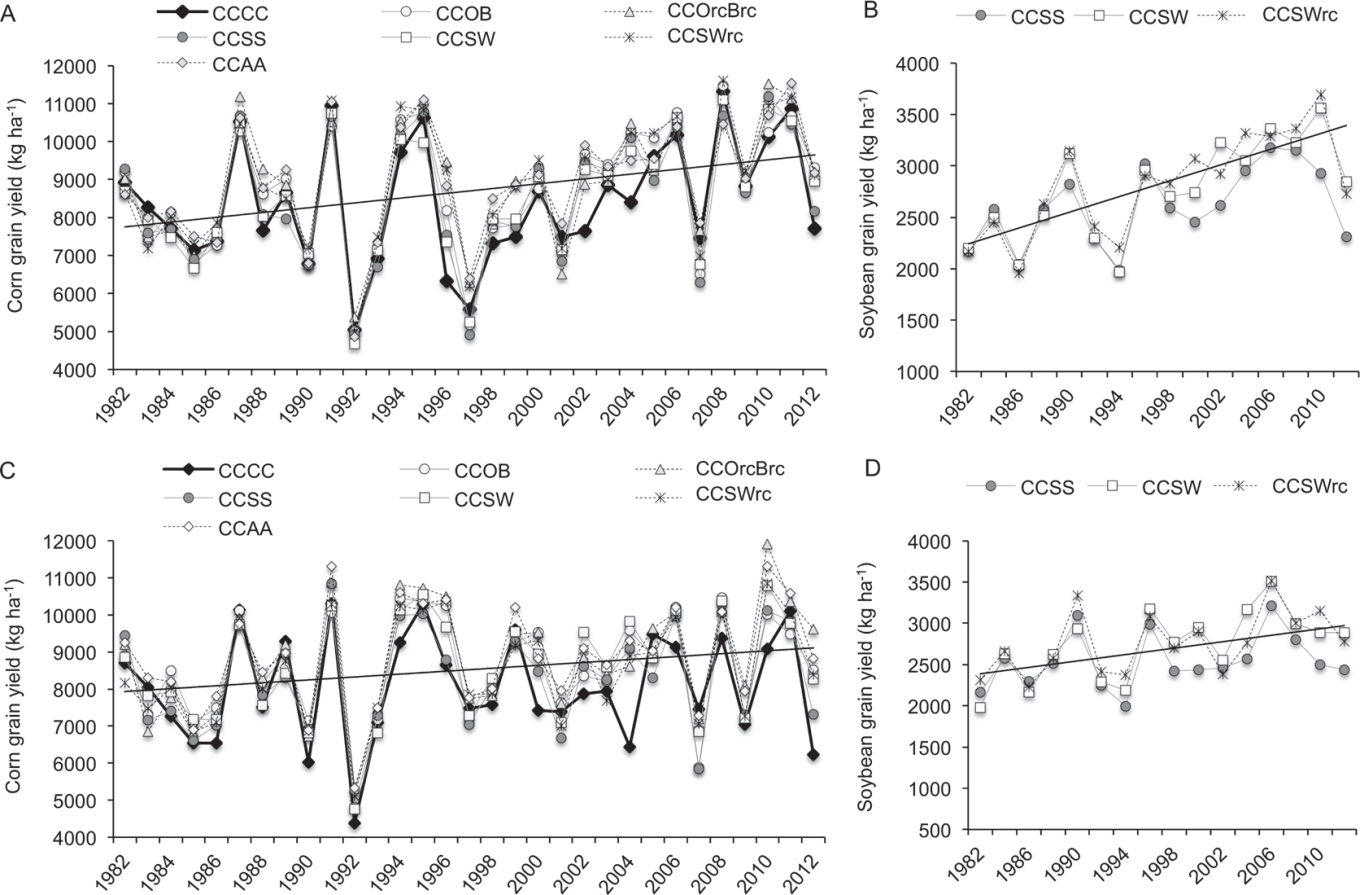
Long term trends in corn and soybean yields under conventional and reduced tillage at Elora. Yields under (A-B) tillage and (C-D) reduced tillage. Each of the 4-year rotation was duplicated 2 years out of phase so that corn and soybean yields were available yearly (n = 31) and biyearly (n = 16) respectively. Each main rotation plot was split into two levels of tillage. LS means (n = 4) are shown for each rotation. Crop abbreviation: C = corn, S = Soybean, O = Oat, B = spring barley, W = Winter wheat, rc = underseeded red clover, A = Alfalfa.

More complex rotations decreased corn and soybean yield lags due to reduced tillage (Table 1). Corn mean yield benefits from including alfalfa and wheat with red clover also increased with less tillage, reaching 650kg ha^-1^ compared to short corn rotations (CCCC, CCSS). Mean trial corn and soybean yields similarly increased from 1982 to 2012 by 28% under tillage (Fig. 2A, B) with lower rates when tillage is reduced (17%) (Fig. 2C, D). Across tillage and rotation treatments, corn yields deviated from the trendline up to ∼41% (Fig. 2A, C) compared to ∼16% in soybean in unfavorable years (Fig. 2B, D).

### Crop diversity lowers risk of crop failure

Short rotations (CCCC and CCSS) consistently had the highest probabilities of corn yields falling in the lower component of the pooled dataset P(dp) in both tillage systems (Table 2). Short rotations also showed high risks of yielding below the 10^th^ percentile (P(ly); <7546.7 kg ha^-1^) (Table 2). Diverse rotation including small grains and forages presented the lowest P(dp) and (P(ly), especially in conventionally tilled systems. Alfalfa in rotations reduced (P(ly)) to only 5.7% and 6.8% in reduced and conventional tillage respectively. Crop diversification also increased the probability of achieving high yields (P(hy); >11321.4 kg ha^-1^) while short rotations consistently had the lowest P(hy) (Table 2). Relay cropping red clover increased the likelihood of yields exceeding the 90^th^ percentile threshold by more than two fold under conventional tillage. Tillage increased P(hy) and decrease P(ly) for all rotations with large tillage effects for continuous corn (CCCC) and smaller effects when forage were introduced into rotations (Table 2). In reduced tillage systems, most rotations except CCOrcBrc (rank #3, 12.1%) and CCAA (rank #4, 11.1%) had no better than a 5% probability of yields above the 90^th^ percentile (Table 2).

**Table 2:** Effect of rotation diversity and tillage on probabilities of obtaining high and low corn yield based on long-term yield distributions.

	Probability of High Yield			Probability of Low Yield						Performance Index		
	P(*yield > 90* ^*th*^ *percentile*)			Downside potential			P(*yield < 10* ^*th*^ *percentile*)					
Rotation	P(hy)	(*p*-value)	Rank	P(dp)	(*p*-value)	Rank	P(ly)	(*p*-value)	Rank	Pi	(*p*-value)	Rank
Tillage												
CCCC	9.4%	(0.3268)	7	82.3%	(0.8912)	11	14.8%	(0.8836)	11	68^###^	(1.0000)	9
CCOB	10.2%***	(0.2142)	6	76.0%	(0.0388)	3	12.8%	(0.6764)	10	79^###^	(0.9212)	6
CCOrcBrc	13.7%**	(0.0262)	1	75.2%**	(0.0136)	2	9.1%	(0.1754)	5	95***	(0.0032)	1
CCSS	9.3%	(0.3426)	8	79.1%	(0.3714)	7	15.1%^#^	(0.9024)	13	68^###^	(1.0000)	9
CCSW	7.2%	(0.7130)	9	80.6%	(0.6548)	8	12.3%	(0.6102)	9	69^###^	(1.0000)	7
CCSWrc	13.2%**	(0.0342)	2	74.9%***	(0.0076)	1	9.0%	(0.1640)	4	95***	(0.0032)	1
CCAA	10.5%	(0.1832)	5	77.5%	(0.1410)	6	6.8%**	(0.0242)	2	86	(0.3912)	5
Reduced Tillage												
CCCC	3.1%^###^	(0.9960)	14	87.5%^###^	(1.0000)	14	20.8%^###^	(0.9984)	14	48^###^	(1.0000)	14
CCOB	5.0%^##^*	(0.9496)	10	80.6%	(0.6548)	8	10.8%	(0.3946)	7	69^###^	(1.0000)	7
CCOrcBrc	12.1%*	(0.0650)	3	77.0%*	(0.0922)	4	7.0%**	(0.0290)	3	91**	(0.0496)	3
CCSS	4.6%^##^	(0.9664)	11	85.4%^###^	(0.9964)	13	15.0%	(0.8942)	12	57^###^	(1.0000)	13
CCSW	4.1%^##^	(0.9828)	13	80.7%	(0.6706)	10	11.7%	(0.5272)	8	60^###^	(1.0000)	12
CCSWrc	4.6%^##^	(0.9664)	11	82.5%^#^	(0.9070)	12	9.8%	(0.2572)	6	64^###^	(1.0000)	11
CCAA	11.1%	(0.1216)	4	77.0%*	(0.0922)	4	5.7%***	(0.0044)	1	91**	(0.0496)	3

Shown are the probabilities (%) of obtaining yields above or below certain thresholds and their respective ordinal ranks based on probability densities from estimated yield distributions. P(hy) = probability of achieving yields above 11321.4 kg ha^-1^; P(dp) = probability that a given treatment falls into the lower component of the polled data; P(ly) = probability of yields falling below 7546.7 kg ha^-1^. Performance indexes (Pi, max = 104) were calculated based on ordinal ranks for each metrics. Statistical significance is based on randomized p-values and indicated by (*, ^#^) 10%, (**, ^##)^ 5%, and (***, ^###^) 1% for metrics that are higher (*) or lower (^#^) than randomized metrics. Crop abbreviations: C = Corn, S = Soybean, A = Alfalfa, W = Winter wheat, O = Oat, B = Spring barley, rc = under seeded red clover.

Performance indexes taking into account all three metrics increased with rotation complexity across tillage treatments (Table 2). Short rotations under reduced tillage had the lowest Pi scores (Table 2) while maximum Pi score were obtained in CCOrcBrc and CCSWrc rotations under conventional tillage. Relay cropping red clover with small grains was instrumental to improve corn performance in tilled systems (CCOB = Pi + 16; CCSW = Pi + 26).

### Crop diversity mitigates yield loss due to hot and dry conditions

**Fig 3:**
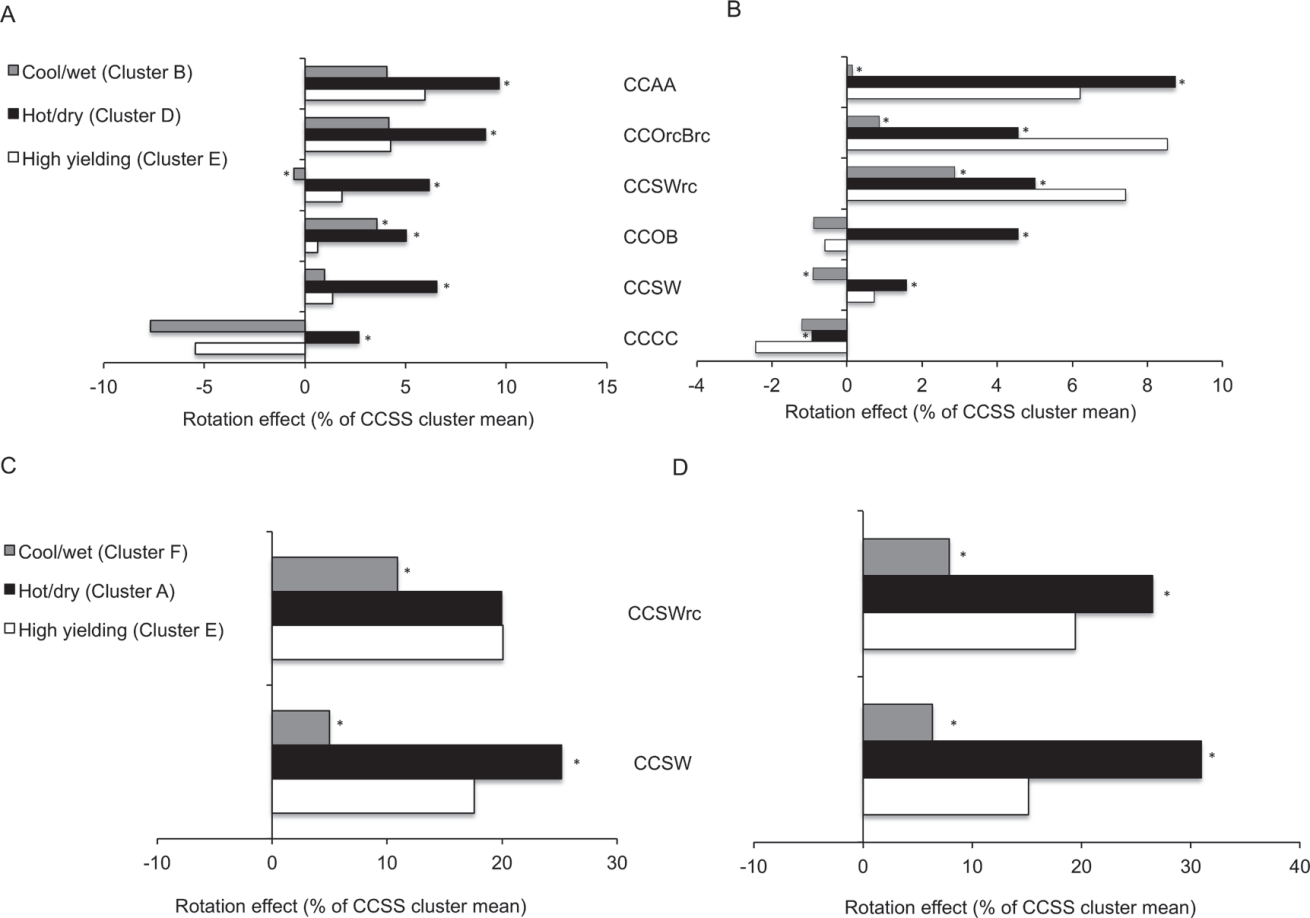
Benefits of diversification under different weather scenarios. (A-B) Corn yields compared to CCSS rotation (%) obtained for selected cluster in (A) reduced tillage and (B) tilled systems. (C-D) Soybean yields compared to CCSS rotation (%) obtained for selected clusters in (C) reduced tillage and (D) tilled systems. Crop abbreviation: C = corn, S = Soybean, O = Oat, B = spring barley, W = Winter wheat, rc = underseeded red clover, A = Alfalfa. (*) significantly different from high yielding years (cluster E) at p = 0.05.

Year cluster D (n = 10) and A (n = 7) grouped the hottest and driest years for corn and soybean respectively (S3, S4 Figs.). Growing seasons were short with significant water deficit and high temperatures during reproductive growth stages, resulting in significantly lower yields than in high yielding years (Cluster E) (S3, S4 Figs., Table 3). Corn yield under all diversification strategies in reduced tillage was increased compared to CCSS rotations when hot and dry conditions occurred (Fig. 3A, S5 Fig.). Benefits of diversification were higher than those observed under favorable conditions. Introducing oat and barley with red clover or alfalfa was particularly beneficial to mitigate droughty conditions (+734 kg ha^-1^ on average, S5 Fig., Fig. 3A). In tilled systems, all rotations but CCSW had higher corn yield than CCSS in dry and hot years (S5 Fig.), the highest yield benefits being obtained from alfalfa in rotation (+8.7% Fig. 3B).

**Table 3:** Corn and soybean mean grain yields per growing season clusters in different tillage systems.

	Corn						Soybean					
	Growing season		Yield (kg ha^-1^)				Growing season		Yield (kg ha^-1^)			
Cluster	(days)		Till		Red. Till		(days)		Till		Red. Till	
A	184.0	a	8667.1	b	8628.6	b	122.9	b	2513.8	c	2661.8	ab
B	172.8	a	8063.7	c	7678.4	c	138.3	a	2834.7	ab	2953.7	a
C	177.0	a	9038.1	a	8681.2	b	126.5	b	2645.5	bc	2669.9	ab
D	159.3	b	8535.3	b	8329.2	bc	129.8	b	2341.4	c	2262.7	c
E	161.4	b	8726.0	ab	9285.5	a	134.7	ab	2904.6	a	2842.7	a
F	N/A		N/A		N/A		144.8	a	2363.4	c	2402.4	b
Mean	167.6		8560.1		8520.0		133.2		2630.4		2682.8	

Growing seasons are defined as the mean number of days from planting to harvest according to historical records for the trial. Clusters were determined according to normalized soil moisture (Hydrus 1D model) and temperature pattern tailored to corn and soybean developmental stages. Least Square Means per tillage treatment followed by the same letter were not significantly different (p = 0.05). Total mean: corn n = 31, soybean n = 16. Till = continuous tillage; Red Till = reduced tillage; (*N/A*) = non applicable.

Similarly for soybean, diversifications of CCSS rotations with wheat led to higher yield benefits in hot and dry conditions than in favorable years (Fig. 3C,D). Although rotation benefits were higher in tilled systems (Fig. 3C,D.) reduction in tillage helped mitigate the impact of dry and hot weather conditions on soybean yields (Table 3).

### Yield benefits of crop diversity are less pronounced in wet and cool weather

Cold and wet growing seasons were grouped in Cluster A (n = 5) and B (n = 6) for corn (S3 Fig.) and Cluster D (n = 7) and F (n = 5) for soybean (S4 Fig.).

Cluster B is of main interest for corn as it grouped years with the coolest, wettest and lowest corn yields over the last 31-years (S3 Fig., Table 3).

On average for both clusters representing wet and cool growing seasons, diversifying a corn-soybean rotation with a forage legume provided 4% and 4.5% corn yield benefits in reduced and conventional tillage respectively (+332, +371 kg ha^-1^, S5 Fig.). Benefits of diversification were lower or similar to the rotation benefits observed under favorable conditions for both crops (Fig. 3); except for CCOB rotations in reduced tillage, which provided higher corn rotation benefits in abnormally cold and wet years than in high yielding years (+ 3.2%, Fig. 3A).

For soybean, Cluster D grouped years that were wet and cold during a time period approximately corresponding with the phenological stage of blooming. No diversification benefits on yields could be detected under reduced tillage when cold, wet conditions occurred during this period. In tilled systems, soybean yields were 24% lower than less complex CCSS rotations (S5 Fig., Cluster D). When cold and wet conditions lasted the whole growing season (Fig. 3C, D, Cluster F), modest rotation benefits were observed.

### Rotation diversity decrease soybean yield variability in “abnormal” years

**Fig 4:**
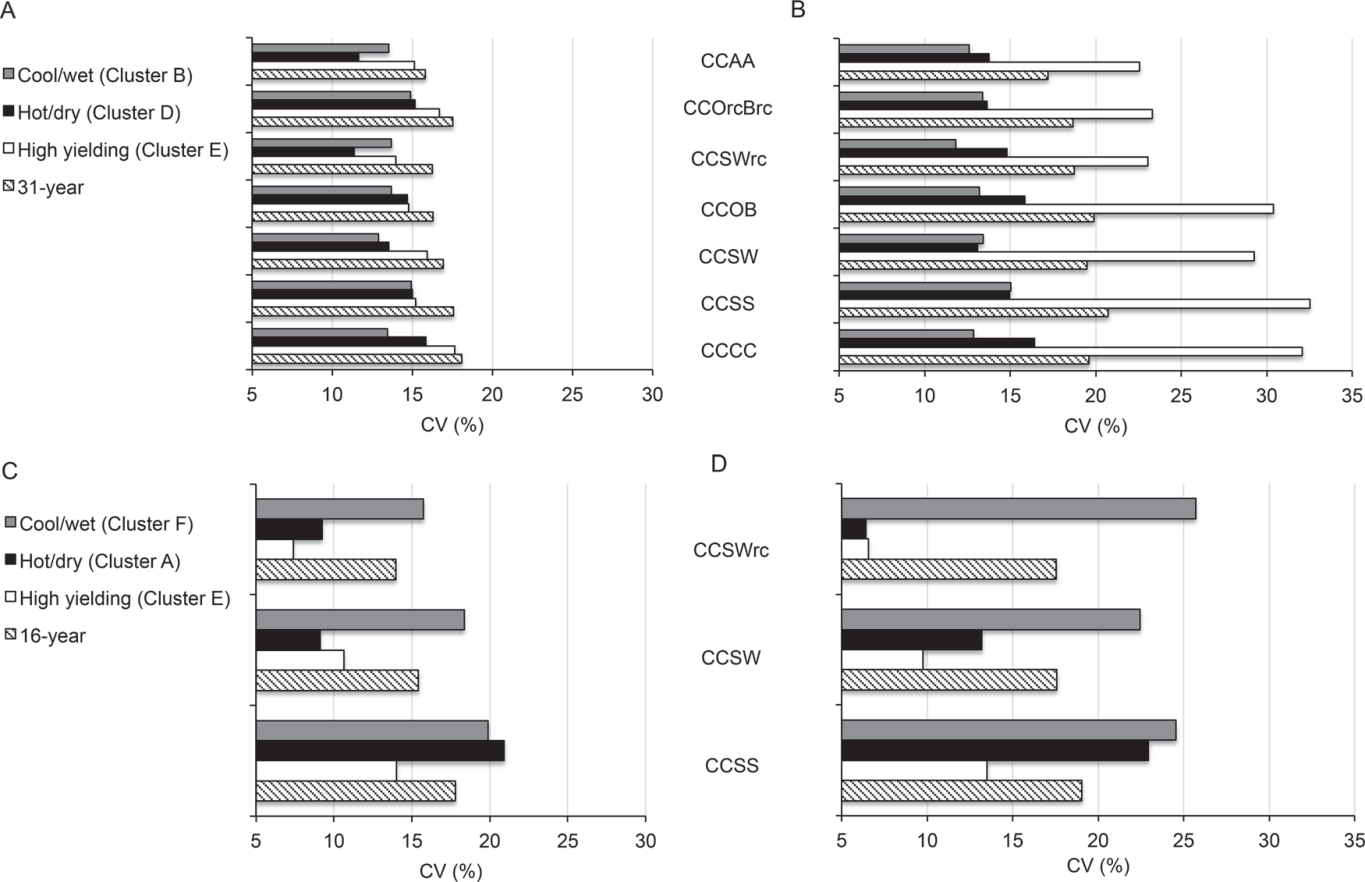
Yield variation under different weather scenarios. (A-B) Coefficient of variation for corn yields (CV, %) obtained for selected cluster and on average across the study period in (A) reduced tillage and (B) tilled systems. (C-D) Coefficient of variation for soybean yields (CV %) obtained for selected cluster in (C) reduced tillge and (D) tilled systems. Crop abbreviation: C = corn, S = Soybean, O = Oat, B = spring barley, W = Winter wheat, rc = underseeded red clover, A = Alfalfa.

Although reduction in tillage decreased yield variability in favorable years, tillage and rotation diversity had no effects on corn yield variation in abnormal hot/dry or cool/wet conditions (Fig. 4A,B). Larger rotation effects could be observed in soybeans (Fig. 4C, D). Diversifying rotations decreased fluctuation in soybean yields due to cool and wet growing conditions up to 4% under reduced tillage (Fig. 4C). Larger increase in yield stability was found when hot and dry conditions occurred (+ 13% till, +11 reduced till). In droughty years, inclusion of wheat and red clover dramatically improved soybean yield stability by 16% compared to CCSS rotations for tilled systems (Fig. 4D).

## Discussion

### Increasing rotation diversity improves yield stability by mitigating hot and dry weather and lowers risk of crop failure

Compared to more diverse rotations, corn-soybean sequences had 1) larger coefficient of variations (Fig. 4); 2) smaller cumulative and mean yields over the course of the experiment (Table 1) and under various weather scenarios (Fig. 3, S5 Fig.); and 3) higher probabilities of achieving low yields under unfavorable conditions (Table 2). Although the magnitude of rotation benefits varied with crops, weather patterns and tillage (Fig. 3), yield stability significantly increased when corn and soybean were integrated into more diverse rotations, especially when legumes were introduced (Fig. 3, Fig. 4). Our findings of increased yield stability support the conclusions of previous studies showing that longer or more diverse rotations improve the temporal stability of grain yields [33,36,37,39,55–57].

Resilience is rooted in the concept of stability, and describes how a system responds under stress [17]. Our study does not investigate systems response to stress during crop development and fluctuations in crop physiological and yield potential status during stress would have to be studied to clearly establish how crop diversity and tillage alter the dynamic of crop stability (ie: resilience, tolerance, resistance). By emphasizing adaptive management strategies, resilience thinking has been proposed as a new way to inform the changes in practices necessary to adapt cropping systems to shifts in resource availability [58]. Future studies are necessary to examine both spatial and temporal aspects of resilience applied to cropping systems.

We clearly show that the effects of crop diversity vary with weather conditions and conclusively attribute larger yield stability in dry years to greater crop diversity (Figs. 3, 4). Previous observations of rotation benefits in droughty or low-yielding environments were often anecdotal or confounded by management practice differences across organic and conventional systems [5,33,34,36,37,39,40,59,60]. Our trial was conventionally managed, with similar fertilizer regime and pest pressure between plots and no effects of such factors on productivity. By attributing higher rotation benefit to growing seasons with hot and dry conditions, the present study emphasizes the potential of system diversity to conserve soil moisture and/or improve plant access to water resources.

It has been shown that genetic diversity can help reduce stress exposure and the risk of crop failure, with significant economic impact for producers [61–64]. To capture changes in exposure to downside potential, assessments needs to go beyond a simple measure of yields and its dispersion [61]. Our non-parametric approaches estimating changes in corn yield probabilities showed that crop diversification strategies increased the probability of harnessing favorable growing conditions and achieving high corn yields while decreasing the likelihood of having abnormally low yields in unfavorable conditions (Table 2). The finite mixture models were useful in approximating many of the structures associated with corn yield distributions, and allowed analysis without using predetermined thresholds and produced straightforward parameter estimates [65–67].

### Tillage practices alter the magnitude of crop diversity benefits in abnormal years

Crop diversification and reduction in tillage had synergistic effects in both crops: less tillage further enhanced rotation benefits (Table 1), yield stability (Fig. 4) and corn yields under unfavorable growing conditions (Fig. 3 A, B). It has been shown that diverse crop rotations are needed to decrease yield lags due to reduced tillage and improve soil properties at this trial [29,34,68,69].

We found high variability in the synergistic effects of rotation and tillage according to weather scenarios (Fig. 3, Fig. 4) and considerable differences between environments have been reported [30,35,40,70–72]. Under favorable conditions, tillage increased the probability of reaching high corn yields for all rotations with larger tillage effects for short rotations (Table 2). Conventional tillage decreased the corn yield penalty due to monoculture (-3%) and increased benefits from relay cropping red clover (+5%) in favorable years (Fig. 3A, B). Conversely, we observed significant increase in corn yields in dry and hot weather in all rotations with reduced tillage (Fig. 3A, B).

Differences in rotation benefits with tillage may be, in part, attributed to variation in soil moisture and temperature associated with changes in residue cover [40,68,73]. Excessive or deficient soil moisture is a significant factor in the relative performance of corn and soybean in different tillage systems [74]. Residues in reduced tilled systems often delay soil warming, planting date and emergence which may have decreased corn yield potential in our short growing seasons when conditions were favorable [29,34,74]. However, less aggressive tillage decreased variations in corn yields in such years (Fig. 4A, B), probably due to buffering effects later in the season. Crop residues may have helped to mitigate the impact of hot and dry weather on corn yields by restricting water loss, delaying soil warming, reducing air temperature at the soil surface and reducing evaporation potential [75–77]. Review articles illustrate that moisture conservation effects of surface residue increase corn and soybean yields in arid environments [74,78].

Reduction in tillage further increased the benefits obtained from inclusion of cereals and forage legumes (S5 Fig.). It also improved soybean yield stability compared to tilled systems under cool and wet conditions (Fig. 4C, D). Although retention of soil moisture may cause problems in the early spring, reduction in tillage help establish macropore continuity which may aid in drainage of excess soil moisture and decrease compaction and anaerobiosis when wet conditions persist during the growing season [79,80].

Corn and soybean responded differently to diversification and tillage practices. Introducing small grains into short corn-soybean rotation was enough to provide substantial benefits on long-term soybean yields and their stability while the effects on corn were mostly associated with the temporal niche provided by small grains for underseeded red clover or alfalfa (Fig. 3, Table 1). Soybean yields were also less responsive than corn yields to changes in tillage practices (S5 Fig.) and less susceptible to yearly variations in weather (Fig. 2, Fig. 4) as shown in previous studies [40]. Finally, soybean and corn had opposite responses to tillage in hot and dry years (Fig. 3). Such distinctions between the two crops may be due to differential response to changes in soil properties associated with diversification and tillage, nutrient and physiological requirements, growth habits, compensation of individual yield components and their impact on crop susceptibility to environmental stresses and potential success of reproduction and grain filling [7,81–83]. Tillage effects on rotation responses for corn and soybeans might also been influenced by odd years that only contained corn comparisons.

### Potential of crop diversification and reduced tillage to improve yields under hot and dry weather through soil improvements

In natural ecosystems, the coexistence of multiple species with similar functions but with different responses to perturbation enhances resilience [84,85]. In our study, the effect of greater temporal and spacial diversity on soil properties associated with soil water storage and plant access to moisture likely minimize the risks of significant crop yield fluctuations in response to different rainfall amount or distribution pattern. Rotation benefits in challenging environments were previously associated with higher soil moisture conservation and percolation in the root zone [36,37] and increase in precipitation use efficiencies [38,40].

Similar management strategies to the one tested here have been shown to improve several soil properties important to build resilient soils [29,34,68,86–88]. Of particular importance to mitigate the effects of variation in precipitation patterns, are the positive direct and indirect effects of reduced tillage, small grain cereal roots and red clover on soil structure, aggregation and organic carbon levels reported for this trial [29,34,89,90], as they all contribute to the soil’s ability to capture and conserve precipitation water [91,92].

Higher aggregate stability has been shown to improve soil permeability, aeration, infiltration rates, and reduce runoff and penetration resistance [86,87,93–95]. More aggregated soil structure also fosters more extensive and deeper root systems [96,97] which may make additional moisture available to the crop. Soils with high levels of organic matter also have higher water-holding capacity, water infiltration rates and permeability [91,95,98,99]. Higher soil moisture in turn may maintain cooler soil temperatures for a longer period after drought onset, decreasing plant water losses. Consistent with organic matter accumulation and aggregate stability profiles previously reported [29,34,89,90], significantly higher rotation benefits were observed in hot and dry years for continuous corn grown as well as more diversified rotations compared to CCSS under reduced tillage (Fig. 4A).

Finally, reduced tillage and more complex rotations provide longer periods without disturbances and abundant living plant roots in the soil to host mycorrhizae or earthworms over a greater duration of time within the crop rotation [86,100–102]. This may allow plants to use water more efficiently [103–105].

### Significance for future production environment in the northern Corn Belt

The yield instability and vulnerability of simple rotations observed in this study may be exacerbated in future years by a number of emerging trends. First, the expected trend towards warmer, drier summers and more variable precipitation patterns in the mid latitude regions [9] may accentuate yield gaps in less diverse rotations. Secondly, predicted increases in yield potential of corn and soybean could also increase crop water requirements [106], which may further exacerbate the effects of shifts in precipitation. The greater sensitivity of both crops to drought with increase in yields observed in the Midwest over the last decades [107] reinforce this argument. Finally, development of the bioeconomy may result in higher crop residue removal, with potentially negative impacts on soil organic matter and thus soil water holding capacity and other soil quality parameters. Short corn-soybean rotations and tillage are also being increasingly reported as 1) less sustainable, 2) less productive and profitable and 3) riskier at the system level than more diverse rotations when a longer time frame is considered [19,28,56,62–64,90]. As a result of these trends, the value of diversifying rotations through introduction of wheat and or forage legumes and adoption of reduced tillage systems could increase in the future. Rotation complexity may provide a systems approach to help adapt agroecosystems to upcoming changes in crop growing conditions while addressing the sustainability issues associated with maintaining yields under increasingly challenging production environments.

## Supporting Information

S1 Code R scripts used for non-parametric analysis of crop yields. (ZIP)

S1 Dataset Complete yields and weather variable dataset used for analysis of the long-term rotation and tillage trial. (ZIP)

S1 Fig Harvested areas of field crops grown in Ontario from 1980–2013. Harvested areas (hectares) of major field crops are shown as % of total harvested area from 1981–2013 [27]. Surface area harvested in hay were not included for clarity.
(TIFF)

S2 Fig Characterization of variation between growing seasons according to crop development. (A-B) Soil volumetric water content during (A) corn and (B) soybean development. (C-D) Biplot of principal component analysis of variation in weather variables during (C) corn and (D) soybean development. Dry and hot (cluster D for corn, cluster A for soybean) and wet and cool (cluster A,B for corn and D,F for soybean) years are shown. Corn and soybean developmental stages were estimated based on crop heat units and planting, flowering and harvesting dates recorded from 1982–2012. Soil volumetric water contents for each year were modeled in the Hydrus 1D environment. Field capacity and permanent wilting points were used to determine the volumetric soil water content at 50% and 25% of available soil water (ASW). Abbreviations: SVWC = Soil Volumetric Water Content. Veg = Vegetative, Flow / Bloom = Flowering/ Blooming, Gr. Fill/ Pod. Fill = Grain/ Pod filling, Mat = Maturation.
(TIF)

S3 Fig Clustering of 31 years of weather data according to corn developmental stages. (A) Heatmap and dendogram of the aggregative structure of normalized data and (B) summary of mean soil water content and temperatures for each cluster. Euclidian distances and Ward linkage functions were used for hierarchical clustering of aggregative data. Abbreviations: SVWC = Soil Volumetric Water Content. Veg = Vegetative, Flow = Flowering, Gr. Fill = Grain filling, Mat = Maturation.
(TIF)

S4 Fig Clustering of 31 years of weather data according to soybean developmental stages. (A) Heatmap and dendogram of the aggregative structure of normalized data and (B) summary of mean soil water content and temperatures for each cluster. Euclidian distances and Ward linkage functions were used for hierarchical clustering of aggregative data. Abbreviations: SVWC = Soil Volumetric Water Content. Veg = Vegetative, Bloom = Blooming, Pod. Fill = Pod filling, Mat = Maturation.
(TIF)

S5 Fig Yield benefits of diversification under different weather scenarios. (A-B) Corn yields compared to CCSS rotation (Δ yield) obtained for each cluster in (A) reduced tillage and (B) tilled systems. (C-D) Soybean yields compared to CCSS rotation (Δ yield) obtained for each cluster in (C) reduced tillage and (D) tilled systems. Crop abbreviation: C = corn, S = Soybean, O = Oat, B = spring barley, W = Winter wheat, rc = underseeded red clover, A = Alfalfa. (*) significantly different from CCSS yields at p = 0.05.
(TIF)

S1 Table Estimated optimal number of components in Normal mixture model. The null hypothesis for the EM-Test of model fit was that a two component Normal mixture model does not provide a statistically significant improvement in fit compared to a Normal distribution. Akaike Information Criterion with small sample correction (AICC) and Bayesian Information Criterion (BIC) were used to compare two components in the mixture model to three (with the caveat they only recover the optimal number of components asymptotically). EM-tests show that two components fitted better than one in all cases while two components provided a lower AICC and BIC in all cases; therefore, we estimated a two-component mixture for all treatments. Crop abbreviations: C = Corn, S = Soybean, A = Alfalfa, W = Winter wheat, O = Oat, B = Spring barley, rc = under seeded red clover.
(DOCX)

S2 TableParameters of the estimated yield distribution mixture models.(μ) mean yields in kg ha^-1^ and (σ2) variance of the upper and lower components of the aggregated (all treatments, n = 434) and treatment-conditional (n = 31) corn yield density distributions. Crop abbreviations: C = Corn, S = Soybean, A = Alfalfa, W = Winter wheat, O = Oat, B = Spring barley, rc = under seeded red clover.(DOCX)

S3 Table Estimated temporal pattern in mean and corn yield variance. *F*-statistic without treatment fixed-effects indicates that including a time trend provides a statistically significant improvement in fit over an intercept-only model while adding treatment effects did not; therefore, we use the parsimonious model without treatment effects (note the qualitative results from the analysis in Table 2 do not change under different trend assumptions). The log-linear regression of non-constant yield variance is based on the empirical heteroscedasticity coefficient procedure of [108] and fails to reject the null hypothesis of homoscedasticity. Therefore, we did not include a correction for non-constant variance and the temporally adjusted (i.e. detrended) yields are re-centered to 2012 based on the estimated time trend.
(DOCX)

S4 Table Probability of a high yield under alternative trend and density estimation assumptions. Trend (1–3) and density (a-b) estimation methods: (1) linear without treatment effects; (2) linear trend estimation with treatment effects; (3) nonparametric local regression trend; (a) mixture of two Normals; (b) nonparametric kernel density estimate
(DOCX)

S5 Table Probability of downside potential under alternative trend and density estimation assumptions. Trend (1–3) and density (a-b) estimation methods: (1) linear without treatment effects; (2) linear trend estimation with treatment effects; (3) nonparametric local regression trend; (a) mixture of two Normals; (b) nonparametric kernel density estimate.
(DOCX)

S6 Table Probability of low yields under alternative trend and density estimation assumptions. Trend (1–3) and density (a-b) estimation methods: (1) linear without treatment effects; (2) linear trend estimation with treatment effects; (3) nonparametric local regression trend; (a) mixture of two Normals; (b) nonparametric kernel density estimate.
(DOCX)

S7 Table Overall performance index under alternative trend and density estimation assumptions. Trend (1–3) and density (a-b) estimation methods: (1) linear without treatment effects; (2) linear trend estimation with treatment effects; (3) nonparametric local regression trend; (a) mixture of two Normals; (b) nonparametric kernel density estimate.
(DOCX)

## References

[1] Kucharik CJ, Serbin SP (2008) Impacts of recent climate on Wisconsin corn and soybean yield trends. Environ Res Lett 10.1088/1748-9326/3/3/034003

[2] Urban D, Roberts MJ, Schlenker W, Lobell DB (2012) Projected temperature changes indicate significant increase in interannual variability of U.S. maize yields. Clim Change 112: 525–533.

[3] Lobell DB, Field CB (2007) Global scale climate—crop yield relationships and the impacts of recent warming. Environ Res Lett 10.1088/1748-9326/2/1/014002.

[4] Schlenker W, Roberts MJ (2009) Nonlinear temperature effects indicate severe damages to U.S. crop yields under climate change. Proc Natl Acad Sci USA 106: 15594–15598. 10.1073/pnas.0906865106 19717432 PMC2747166

[5] Porter JR, Semenov MA (2005) Crop responses to climatic variation. Phil Trans R Soc B 360: 2021–2035 16433091 10.1098/rstb.2005.1752 PMC1569569

[6] Southworth J, Randolph JC, Habeck M, Doering OC, Pfeifer RA, et al (2000) Consequences of future climate change and changing climate variability on maize yields in the midwestern United States. Agric Ecosyst Environ 82: 139–158.

[7] Almaraz JJ, Mabood F, Zhou X, Gregorich EG, Smith DL (2008) Climate change, weather variability and corn yield at a higher latitude locale: Southwestern Quebec. Clim Change 88: 187–197.

[8] Dixon BL, Hollinger SE, Garcia P (1994) Estimating corn yield response models to predict impacts of climate change. J Agr Resour Econ 19: 58–68.

[9] Intergovernmental Panel on Climate Change (2013) The physical science basis. Contribution of Working Group I to the Fifth Assessment Report of the Intergovernmental Panel on Climate Change. Cambridge, United Kingdom and New York: Cambridge University Press. 1535 p.

[10] Hatfield JL, Cruse RM, Tomer MD (2013) Convergence of agricultural intensification and climate change in the Midwestern United States: Implications for soil and water conservation. Mar Freshw Res 64: 423–435.

[11] Hatfield JL, Boote KJ, Kimball BA, Ziska LH, Izaurralde RC, et al (2011) Climate impacts on agriculture: Implications for crop production. Agron J 103: 351–370.

[12] Tilman D, Reich PB, Knops JMH (2006) Biodiversity and ecosystem stability in a decade-long grassland experiment. Nature 441: 629–632. 16738658 10.1038/nature04742

[13] Tilman D, Reich PB, Knops JMH (2006) Biodiversity and ecosystem stability in a decade-long grassland experiment. Nature 441: 629–632. 16738658 10.1038/nature04742

[14] Holling C (1973) Resilience and stability of ecological systems. Annu Rev Ecol Syst 4: 1–23.

[15] McNaughton JS (1978) Stability and diversity of ecological communities. Nature 274: 251–253.

[16] Naeem S (2002) Biodiversity: Biodiversity equals instability? Nature 416: 23–24. 11882872 10.1038/416023a

[17] Harrison GW (1979) Stability under environmental stress: Resistance, resilience, persistence, and variability. Am Nat 113: 659–669.

[18] Lin B (2011) Resilience in agriculture through crop diversification: Adaptive management for environmental change. Bioscience 61: 183–193.

[19] Davis AS, Hill JD, Chase CA, Johanns AM, Liebman M (2012) Increasing cropping system diversity balances productivity, profitability and environmental health. PLoS One 10.1371/journal.pone.0047149 PMC3468434 23071739

[20] Gaudin AC, Westra S, Loucks CE, Janovicek K, Martin RC, et al (2013) Improving resilience of northern field crop systems using inter-seeded red clover. Agronomy 3: 148–180.

[21] Liebman M, Helmers MJ, Schulte LA, Chase CA (2013) Using biodiversity to link agricultural productivity with environmental quality: Results from three field experiments in Iowa. Renew Agric Food Syst 28: 115–128.

[22] Côté IM, Darling ES (2010) Rethinking ecosystem resilience in the face of climate change. PLoS Biol 10.1371/journal.pbio.1000438 PMC2910654 20668536

[23] Biggs R, Schlüter M, Biggs D, Bohensky EL, BurnSilver S, et al (2012) Toward principles for enhancing the resilience of ecosystem services. Annu Rev Environ Resour 37: 421–448.

[24] Carpenter S, Arrow K, Barrett S, Biggs R, Brock W, et al (2012) General resilience to cope with extreme events. Sustainability 4: 3248–3259.

[25] Altieri M, Nicholls CI (2013) The adaptation and mitigation potential of traditional agriculture in a changing climate. Clim Change 10.1007/s10584-013-0909-y

[26] Di Falco S, Chavas J (2008) Rainfall shocks, resilience, and the effects of crop biodiversity on agroecosystem productivity. Land Econ 84: 83–96.

[27] OMAFRA (2013) Field crops statistics. http://www.omafra.gov.on.ca/english/stats/crops/. Accessed 2014 Sep 15.

[28] Meyer-Aurich A, Janovicek K, Deen W, Weersink A (2006) Impact of tillage and rotation on yield and economic performance in corn-based cropping systems. Agron J 98: 1204–1212.

[29] Munkholm LJ, Heck RJ, Deen B (2013) Long-term rotation and tillage effects on soil structure and crop yield. Soil Tillage Res 127: 85–91.

[30] Singer J, Cox WJ (1998) Corn growth and yield under different crop rotation, tillage, and management systems. Crop Sci 38: 996–1003.

[31] Smith RG, Gross KL, Robertson GP (2008) Effects of crop diversity on agroecosystem function: Crop yield response. Ecosystems 11: 355–366.

[32] Stanger TF, Lauer JG (2008) Corn grain yield response to crop rotation and nitrogen over 35 years. Agron J 100: 643–650.

[33] Peterson TA, Varvel GE (1989) Crop yield as affected by rotation and nitrogen rate. I. Soybean. Agron J 81: 727–731.

[34] Raimbault BA, Vyn TJ (1991) Crop rotation and tillage effects on corn growth and soil structural stability. Agron J 985: 979–985.

[35] Katsvairo TW, Cox WJ (2000) Tillage ϫ rotation ϫ management interactions in corn. Agron J 92: 493–500.

[36] Lotter DW, Seidel R, Liebhardt W (2000) The performance of organic and conventional cropping systems in an extreme climate year. Am J Alter Agr 18: 1–9.

[37] Pimentel D, Hepperly P, Hanson J, Douds D, Seidel R (2005) Environmental, energetic, and economic comparisons of organic and conventional farming systems. Bioscience 55: 573–582.

[38] Varvel GE (1994) Monoculture and rotation system effects on precipitation use efficiency of corn. Agron J 86: 204–208.

[39] Yamoah CF, Francis CA, Varvel GE, Waltman WJ (1998) Weather and management impact on crop yield variability in rotations. J Prod Agric 11: 219–224.

[40] Wilhelm WW, Wortmann CS (2004) Tillage and rotation interactions for corn and soybean grain yield as affected by precipitation and air temperature. Agron J 96: 425–432.

[41] Soil Classification Working Group (1998) The canadian system of soil classification. Agriculture and Agri-Food Canada Publisher 187p

[42] IUSS Working Group WRB (2006) World reference base for soil resources. World Soil Resources Reports No. 103. FAO, Rome.

[43] OMAFRA (2013) Agronomy guide for field crops. Available: http://www.omafra.gov.on.ca/english/crops/pub811/9fertilizer.htm. Accessed 2014 Mar 26.

[44] Van Genuchten MT (1980) A closed-form equation for predicting the hydraulic conductivity of unsaturated soils. Soil Sci Soc Am J 44: 892–898.

[45] Mualem Y (1976) A new model for predicting the hydraulic conductivity of unsaturated porous media. Water Resour Res 12: 513–522.

[46] Feddes RA, Zaradny H (1978) Model for simulating soil-water content considering evapotranspiration—Comments. J Hydrol 37: 393–397.

[47] Wesseling JG, Elbers JA, Kabat P, Van den Broek BJ (1991) SWATRE: Instructions for input. Internal Note, Winand Staring Centre, Wageningen, the Netherlands.

[48] Hooyer M (2012) Identification of soil moisture deficits influencing genotype-by-environment interactions in Maize (Zea Mays L.). MSc Thesis, The University of Guelph. Available https://atrium.lib.uoguelph.ca/xmlui/handle/10214/4669. Accessed 2014 Jun 2.

[49] Everitt BS, Landau S, Leese M, Stahl D (2011) Hierarchical clustering. In: Cluster Analysis, 5th Edition Chichester: John Wiley & Sons pp. 71–110.

[50] De Mendiburu F (2014) Statistical Procedures for Agricultural Research. R package version 1.2–1. Available http://cran.rproject.org/web/packages/agricolae/index.html. Accessed 2014 March 20.

[51] Fujikoshi Y, Ulyanov V V., Shimizu R (2010) Principal component analysis. In: Multivariate Statistics. Hoboken: John Wiley & Sons pp 283–311.

[52] Chen J, Li P (2009) Hypothesis test for normal mixture models: The EM approach. Ann Stat 37: 2523–2542.

[53] Dempster AP, Laird NM, Rubin DB (1977) Maximum likelihood from incomplete data via the EM algorithm. J R Stat Soc Ser B 39: 1–38.

[54] Givens GH, Hoeting JA (2012) Bootstrapping. In: Computational Statistics. Hoboken: John Wiley & Sons pp 287–321.

[55] Varvel GE (2000) Crop rotation and nitrogen effects on normalized grain yields in a long-term study. Agron J 92: 938–941.

[56] Grover KK, Karsten HD, Roth GW (2009) Corn grain yields and yield stability in four long-term cropping systems. Agron J 101: 940–946.

[57] Smolik JD, Dobbs TL, Rickerl DH (1995) The relative sustainability of alternative, conventional, and reduced-till farming systems. Am J Altern Agric 10: 25–35.

[58] Benson MH, Craig RK (2014) The End of Sustainability. Soc Nat Resour 27: 777–782.

[59] Porter PM, Lauer JG, Lueschen WE, Ford JH, Hoverstad TR, et al (1997) Environment affects the corn and soybean rotation effect. Agron J 89: 441–448.

[60] Lockeretz W, Shearer G, Kohl DH (1981) Organic farming in the corn belt. Science 211: 540–547. 17840931 10.1126/science.211.4482.540

[61] Di Falco S (2006) Crop genetic diversity, farm productivity and the management of environmental risk in rainfed agriculture. Eur Rev Agri Econ 33: 289–314

[62] Yamoah C, Walters D, Shapiro C, Francis C, Hayes M. (2000) Standardized precipitation index and nitrogen rate effects on crop yields and risk distribution in maize. Agric Ecosyst Environ 80: 113–120.

[63] Zentner R, Lafond G, Derksen D, Campbell C (2002) Tillage method and crop diversification: effect on economic returns and riskiness of cropping systems in a thin black Chernozem of the canadian prairies. Soil Tillage Res 67: 9–21.

[64] Helmers G, Yamoah CF, Varvel GE (2001) Separating the impacts of crop diversification and rotations on risk. Agron J 93: 1337–1340.

[65] Goodwin BK, Roberts MC, Coble KH (2000) Measurement of price risk in revenue insurance: Implications of distributional assumptions. J Agric Resour Econ 25:195–214.

[66] Woodard JD, Sherrick BJ (2011) Estimation of mixture models using cross-validation optimization: Implications for crop yield distribution modeling. Am J Agric Econ 93: 968–982.

[67] Böhning D, Hennig C, McLachlan GJ, McNicholas PD (2014) The 2^nd^ special issue on advances in mixture models. Comput Stat Data Anal 71: 1–2.

[68] Drury CF, Tan CS, Welacky TW, Oloya TO, Hamill AS, et al (1999) Red clover and tillage influence on soil temperature, water content, and corn emergence. Agron J 91: 101–108.

[69] Drury CF, Tan CS, Reynolds WD, Welacky TW, Weaver SE, et al (2003) Impacts of zone tillage and red clover on corn performance and soil physical quality. Soil Sci Soc Am J 67: 867–877.

[70] Griffith DR, Kladivko EJ, Mannering J V, West TD, Parsons SD (1988) Long-term tillage and rotation effects on corn growth and yield on high and low organic matter, poorly drained soils. Agron J 80: 599–605.

[71] Karlen DL, Berry EC, Colvin TS, Kanwar RS (1991) Twelve‐year tillage and crop rotation effects on yields and soil chemical properties in northeast Iowa. Commun Soil Sci Plant Anal 22: 19–20.

[72] Gentry LF, Ruffo ML, Below FE (2013) Identifying factors controlling the continuous corn yield penalty. Agron J 105: 295–303.

[73] Johnson MD, Lowery B (1985) Effect of three conservation tillage practices on soil temperature and thermal properties. Soil Soc Sci Am J 49: 1547–1552.

[74] DeFelice MS, Carter PR, Mitchell SB (2006) Influence of tillage on corn and soybean yield in the United States and Canada. Crop Management 10.1094/CM-2006-0626-01-RS

[75] Horton R, Bristow KL, Kluitenberg GJ, Sauer TJ (1996) Crop residue effects on surface radiation and energy balance. Review. Theor Appl Climatol 54: 27–37.

[76] Mitchell J, Singh P, Wallender W, Munk D, Wroble J, et al (2012) No-tillage and high-residue practices reduce soil water evaporation. Calif Agric 66: 55–61.

[77] Blevins RL, Smith MS, Thomas GW, Frye WW (1983) Influence of conservation tillage on soil properties. J Soil Water Conserv 38: 301–305.

[78] Toliver DK, Larson JA, Roberts RK, English BC, De La Torre Ugarte DG, et al (2012) Effects of no-till on yields as influenced by crop and environmental factors. Agron J 104: 530–541.

[79] Seta AK, Blevins RL, Frye WW, Barfield BJ (1993) Reducing soil erosion and agricultural chemical losses with conservation tillage. J Environ Qual 22: 661–665.

[80] VandenBygaart AJ, Protz R, Tomlin AD (1999) Changes in pore structure in a no-till chronosequence of silt loam soils, southern Ontario. Can J Soil Sci 79: 149–160.

[81] Runge ECA (1968) Effects of rainfall and temperature interactions during the growing season on corn yield. Agron J 60: 503–507.

[82] Runge ECA, Odell RT (1960) The relation between precipitation, temperature, and the yield of soybeans on the Agronomy South Farm, Urbana, Illinois. Agron J 52: 245–247.

[83] Hu Q, Buyanovsky G (2003) Climate effects on corn yield in Missouri. J Appl Meteorol 42: 1626–1635.

[84] Walker B (1995) Conserving biological diversity through ecosystem resilience. Conserv Biol 9: 747–752.

[85] Elmqvist T, Folke C, Nyström M, Peterson G, Bengtsson J, et al (2003) Response diversity, ecosystem change, and resilience. Front Ecol Environ 1: 488–494.

[86] Katsvairo T, Cox WJ, Van Es H (2002) Tillage and rotation effects on soil physical characteristics. Agron J 94: 299–304.

[87] Carter MR, Kunelius HT (1993) Effect of undersowing barley with annual ryegrasses or red clover on soil structure in a barley-soybean rotation. Agric Ecosyst Environ 43: 245–254.

[88] Dapaah HK, Vyn TJ (1998) Nitrogen fertilization and cover crop effects on soil structural stability and corn performance. Commun Soil Sci Plant Anal 29: 2557–2569.

[89] Mueller L, Kay BD, Deen B, Hu C, Zhang Y, et al (2009) Visual assessment of soil structure: Part II. Implications of tillage, rotation and traffic on sites in Canada, China and Germany. Soil Tillage Res 103: 188–196.

[90] Meyer Aurich A, Weersink A, Janovicek K, Deen B (2006) Cost efficient rotation and tillage options to sequester carbon and mitigate GHG emissions from agriculture in Eastern Canada. Agric Ecosyst Environ 117: 119–127.

[91] Hudson B (1994) Soil organic matter and available water capacity. J Soil Water Conserv 49: 189–194.

[92] Franzluebbers A. (2002) Water infiltration and soil structure related to organic matter and its stratification with depth. Soil Tillage Res 66: 197–205.

[93] Morin J, Benyamini Y, Michaeli A (1981) The effect of raindrop impact on the dynamics of soil surface crusting and water movement in the profile. J Hydrol 52: 321–335.

[94] Angers DA, Mehuys GR (1993) Aggregate stability to water. In: Carter MR, editor. Soil sampling and methods of analysis. Boca Raton: Lewis Publishers pp. 651–658.

[95] Carter MR (2002) Soil quality for sustainable land management: Organic matter and aggregation interactions that maintain soil function. Agron J 94: 38–47.

[96] Tisdall J, Oades J (1979) Stabilization of soil aggregates by the root systems of ryegrass. Aust J Soil Res 17: 429–441.

[97] Six J, Elliott ET, Paustian K (1999) Aggregate and soil organic matter dynamics under conventional and no-Tillage systems. Soil Sci Soc Am J 63: 350–1358.

[98] Haynes RJ, Swift RS, Stephen RC (1991) Influence of mixed cropping rotations (pasture-arable) on organic matter content, water stable aggregation and clod porosity in a group of soils. Soil Tillage Res 19: 77–87.

[99] Yaacob O, Blair GJ (1981) Effect of legume cropping and organic matter accumulation on the infiltration rate and structure stability of a granite soil under a simulated topical environment. Plant Soil 60: 11–20.

[100] Jansa J, Mozafar A, Anken T, Ruh R, Sanders IR, et al (2002) Diversity and structure of AMF communities as affected by tillage in a temperate soil. Mycorrhiza 12: 225–234. 12375133 10.1007/s00572-002-0163-z

[101] Deguchi S, Shimazaki Y, Uozumi S, Tawaraya K, Kawamoto H, et al (2007) White clover living mulch increases the yield of silage corn via arbuscular mycorrhizal fungus colonization. Plant Soil 291: 291–299.

[102] Lehman RM, Taheri WI, Osborne SL, Buyer JS, Douds DD (2012) Fall cover cropping can increase arbuscular mycorrhizae in soils supporting intensive agricultural production. Appl Soil Ecol 61: 300–304.

[103] Tobar RM, Azcon R, Barea JM (1994) The improvement of plant N acquisition from an ammonium-treated, drought-stressed soil by the fungal symbiont in arbuscular mycorrhizae. Mycorrhiza 4: 105–108.

[104] Al-Karaki GN, Clark RB (1998) Growth, mineral acquisition, and water use by mycorrhizal wheat grown under water stress. J Plant Nutr 21: 263–276.

[105] Lazcano C, Barrios-Masias FH, Jackson LE (2014) Arbuscular mycorrhizal effects on plant water relations and soil greenhouse gas emissions under changing moisture regimes. Soil Biol Biochem 74: 184–192.

[106] Richards R (2000) Selectable traits to increase crop photosynthesis and yield of grain crops. J Exp Bot 51: 447–458. 10938853 10.1093/jexbot/51.suppl_1.447

[107] Lobell DB, Roberts MJ, Schlenker W, Braun N, Little BB, et al (2014) Greater sensitivity to drought accompanies maize yield increase in the U.S. Midwest. Science 344: 516–519. 10.1126/science.1251423 24786079

[108] Harri A, Coble KH, Ker AP, Goodwin BJ (2011) Relaxing heteroscedasticity assumptions in area-yield crop insurance rating. Amer J Agr Econ 93: 707–717.

