# Supplementary figures and images for: Increasing Crop Diversity Mitigates Weather Variations and Improves Yield Stability
Source: PLoS One. 2015 Feb 6;10(2):e0113261. doi: 10.1371/journal.pone.0113261 (PMC4320064; doi:10.1371/journal.pone.0113261)

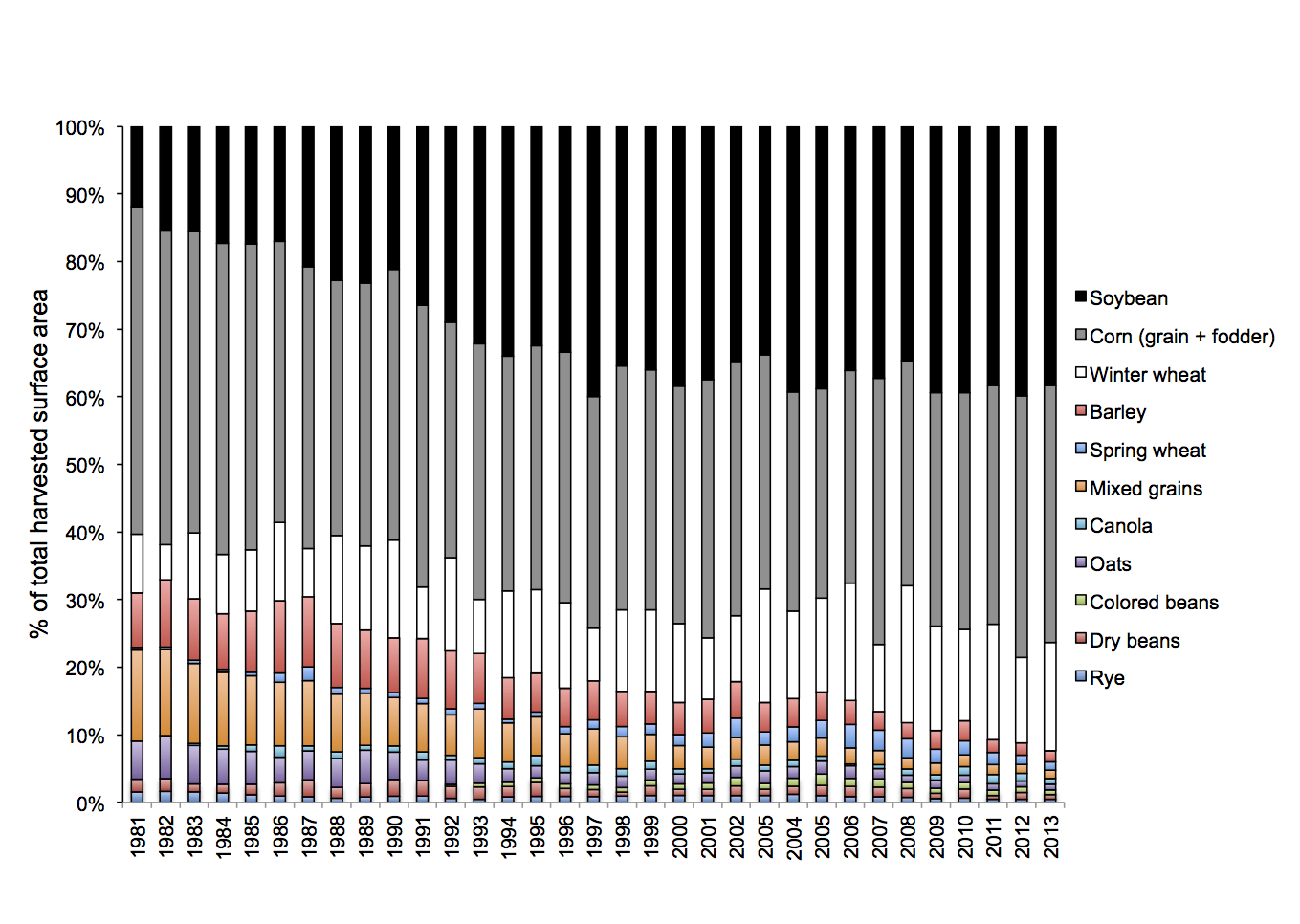

Supplement: S1 Fig — Harvested areas (hectares) of major field crops are shown as % of total harvested area from 1981–2013 [27]. Surface area harvested in hay were not included for clarity. (TIFF) [file pone.0113261.s003.tiff]

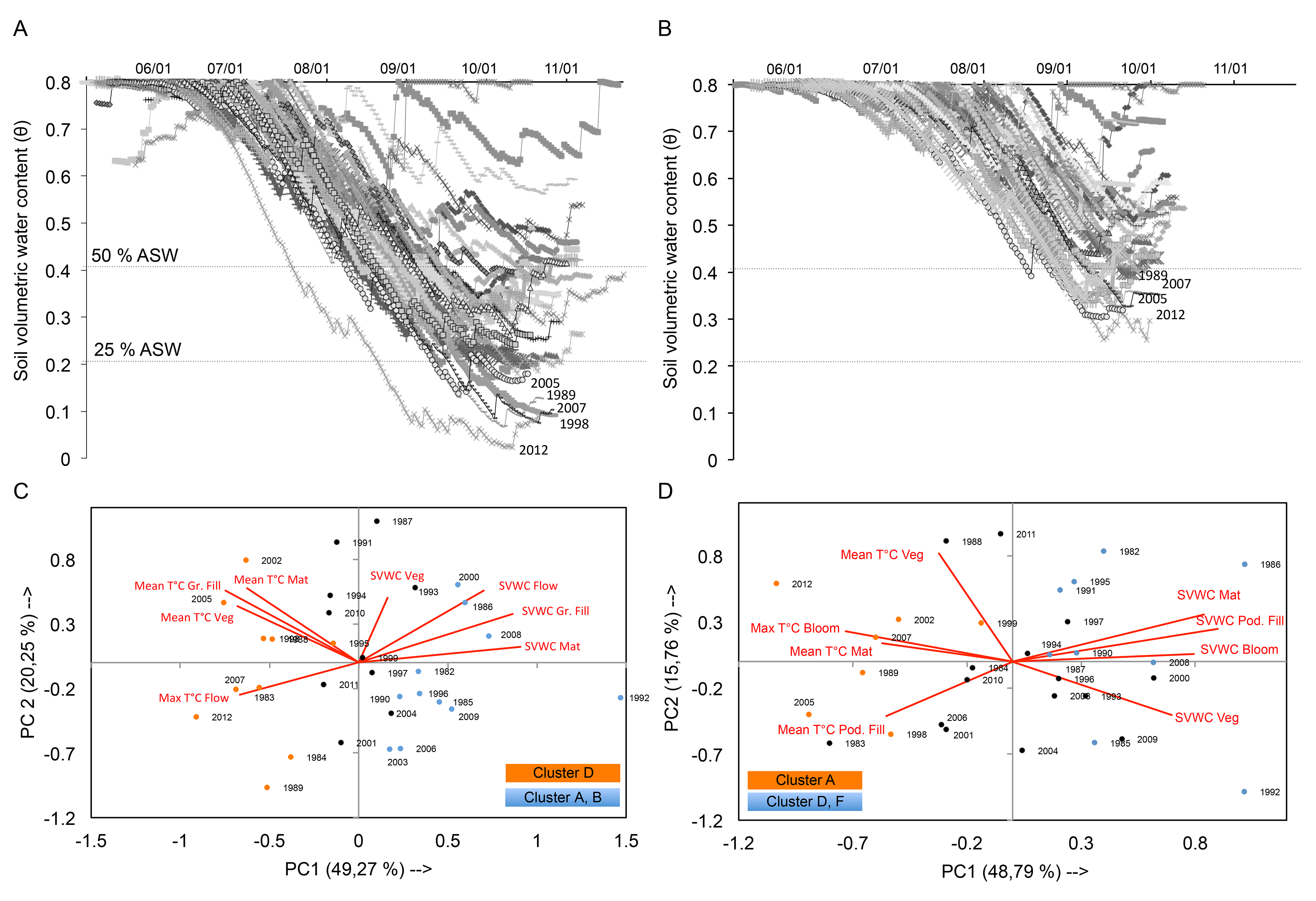

Supplement: S2 Fig — (A-B) Soil volumetric water content during (A) corn and (B) soybean development. (C-D) Biplot of principal component analysis of variation in weather variables during (C) corn and (D) soybean development. Dry and hot (cluster D for corn, cluster A for soybean) and wet and cool (cluster A,B for corn and D,F for soybean) years are shown. Corn and soybean developmental stages were estimated based on crop heat units and planting, flowering and harvesting dates recorded from 1982–2012. Soil volumetric water contents for each year were modeled in the Hydrus 1D environment. Field capacity and permanent wilting points were used to determine the volumetric soil water content at 50% and 25% of available soil water (ASW). Abbreviations: SVWC = Soil Volumetric Water Content. Veg = Vegetative, Flow / Bloom = Flowering/ Blooming, Gr. Fill/ Pod. Fill = Grain/ Pod filling, Mat = Maturation. (TIF) [file pone.0113261.s004.tif]

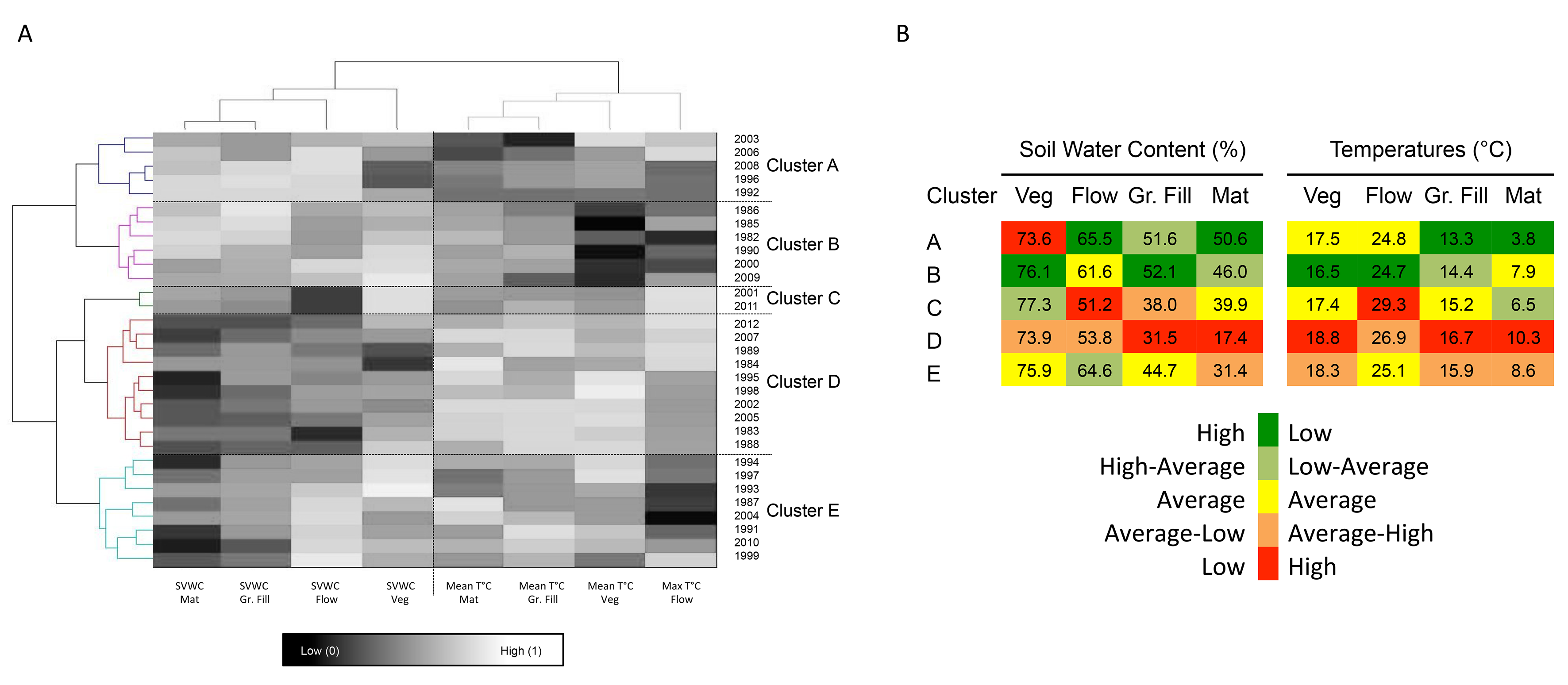

Supplement: S3 Fig — (A) Heatmap and dendogram of the aggregative structure of normalized data and (B) summary of mean soil water content and temperatures for each cluster. Euclidian distances and Ward linkage functions were used for hierarchical clustering of aggregative data. Abbreviations: SVWC = Soil Volumetric Water Content. Veg = Vegetative, Flow = Flowering, Gr. Fill = Grain filling, Mat = Maturation. (TIF) [file pone.0113261.s005.tif]

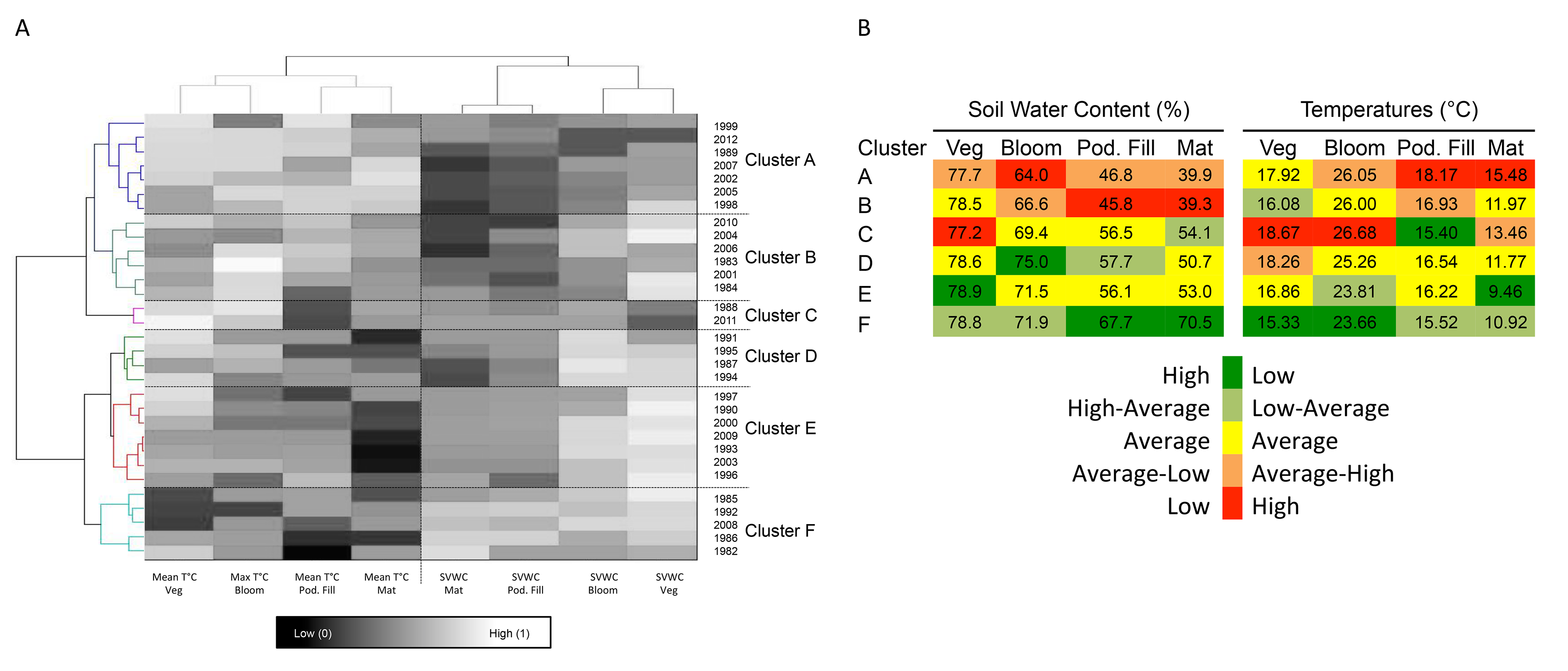

Supplement: S4 Fig — (A) Heatmap and dendogram of the aggregative structure of normalized data and (B) summary of mean soil water content and temperatures for each cluster. Euclidian distances and Ward linkage functions were used for hierarchical clustering of aggregative data. Abbreviations: SVWC = Soil Volumetric Water Content. Veg = Vegetative, Bloom = Blooming, Pod. Fill = Pod filling, Mat = Maturation. (TIF) [file pone.0113261.s006.tif]

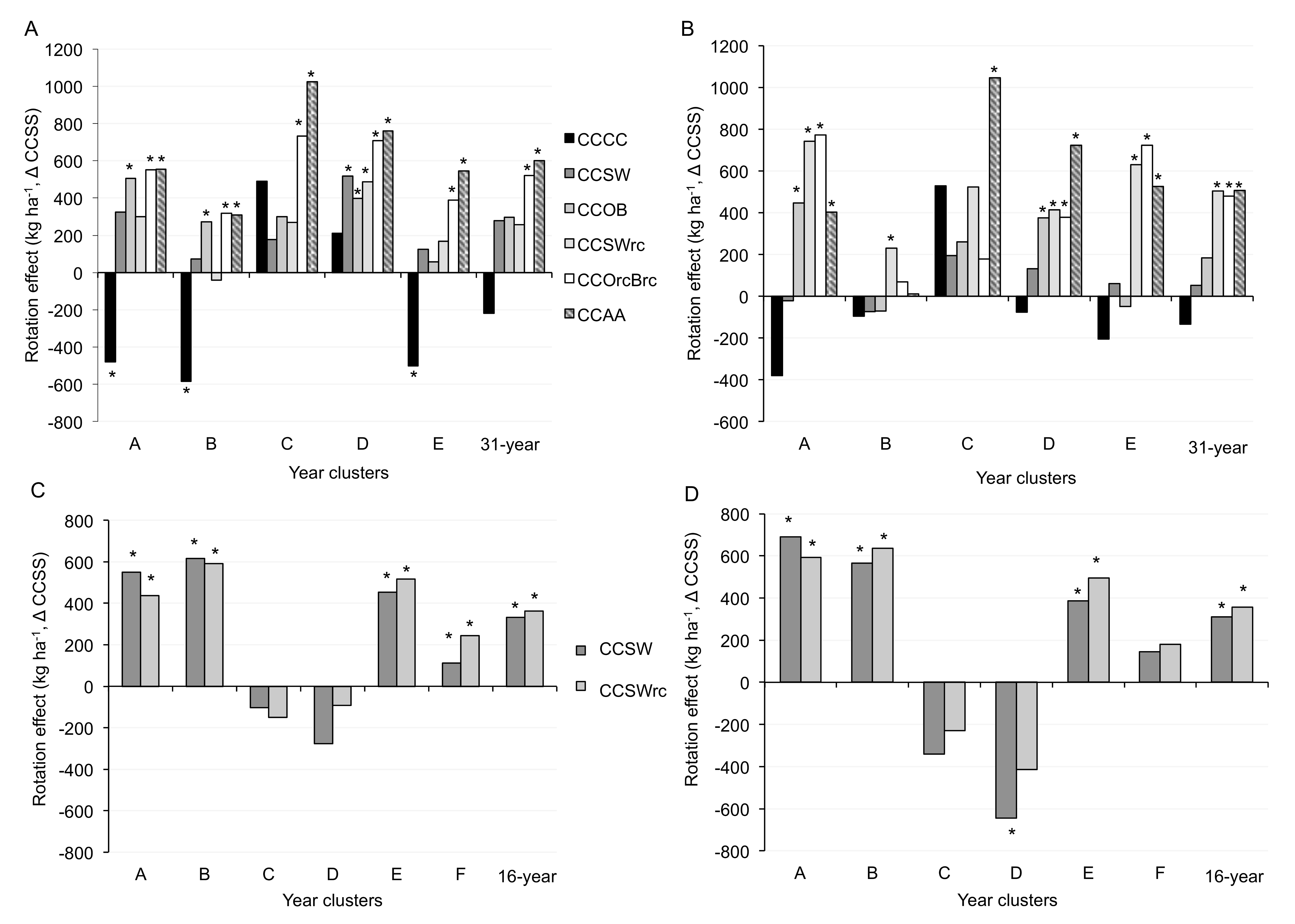

Supplement: S5 Fig — (A-B) Corn yields compared to CCSS rotation (Δ yield) obtained for each cluster in (A) reduced tillage and (B) tilled systems. (C-D) Soybean yields compared to CCSS rotation (Δ yield) obtained for each cluster in (C) reduced tillage and (D) tilled systems. Crop abbreviation: C = corn, S = Soybean, O = Oat, B = spring barley, W = Winter wheat, rc = underseeded red clover, A = Alfalfa. (*) significantly different from CCSS yields at p = 0.05. (TIF) [file pone.0113261.s007.tif]
