# Supplementary material for: Increasing Crop Diversity Mitigates Weather Variations and Improves Yield Stability
Source: PLoS One. 2015 Feb 6;10(2):e0113261. doi: 10.1371/journal.pone.0113261 (PMC4320064; doi:10.1371/journal.pone.0113261)
Supplement: S1 Table — The null hypothesis for the EM-Test of model fit was that a two component Normal mixture model does not provide a statistically significant improvement in fit compared to a Normal distribution. Akaike Information Criterion with small sample correction (AICC) and Bayesian Information Criterion (BIC) were used to compare two components in the mixture model to three (with the caveat they only recover the optimal number of components asymptotically). EM-tests show that two components fitted better than one in all cases while two components provided a lower AICC and BIC in all cases; therefore, we estimated a two-component mixture for all treatments. Crop abbreviations: C = Corn, S = Soybean, A = Alfalfa, W = Winter wheat, O = Oat, B = Spring barley, rc = under seeded red clover. (DOCX) [file pone.0113261.s008.docx]

**Supporting Information Table S1.** Estimated optimal number of components in Normal mixture model

|  |  | EM-Test | | Akaike Information Criterion (AICC) | | Bayesian Information Criterion (BIC) | |
| --- | --- | --- | --- | --- | --- | --- | --- |
| Treatment | Observations | EM-Stat | *p­*-Value | *J=*2 | *J*=3 | *J=*2 | *J*=3 |
| All treatments | 434 | 278.64 | 0.00000*** | 8591.5 | 8620.9 | 8881.5 | 8928.5 |
| *Tillage* | |  |  |  |  |  |  |
| CCCC | 31 | 25.83 | 0.00000*** | 622.1 | 636.0 | 651.6 | 671.3 |
| CCOB | 31 | 25.15 | 0.00000*** | 632.6 | 646.6 | 645.6 | 665.2 |
| CCOrcBrc | 31 | 31.55 | 0.00000*** | 630.2 | 644.2 | 630.2 | 649.8 |
| CCSS | 31 | 22.62 | 0.00001*** | 634.9 | 648.9 | 628.0 | 647.6 |
| CCSW | 31 | 40.5 | 0.00000*** | 622.8 | 636.8 | 640.8 | 660.4 |
| CCSWrc | 31 | 28.61 | 0.00000*** | 627.0 | 641.0 | 638.7 | 658.3 |
| CCAA | 31 | 32.87 | 0.00000*** | 615.1 | 629.1 | 645.6 | 665.2 |
| *Reduced tillage* | |  |  |  |  |  |  |
| CCCC | 31 | 18.74 | 0.00009*** | 624.8 | 638.8 | 632.7 | 652.4 |
| CCOB | 31 | 21.26 | 0.00002*** | 620.5 | 634.5 | 622.7 | 642.3 |
| CCOrcBrc | 31 | 32.74 | 0.00000*** | 619.0 | 632.9 | 656.8 | 676.5 |
| CCSS | 31 | 23.98 | 0.00001*** | 627.6 | 641.5 | 649.9 | 669.5 |
| CCSW | 31 | 28.07 | 0.00000*** | 621.0 | 634.9 | 648.7 | 668.3 |
| CCSWrc | 31 | 25.28 | 0.00000*** | 619.4 | 633.4 | 613.0 | 632.6 |
| CCAA | 31 | 17.94 | 0.00013*** | 619.1 | 633.0 | 643.6 | 663.2 |
