# Supplementary material for: Increasing Crop Diversity Mitigates Weather Variations and Improves Yield Stability
Source: PLoS One. 2015 Feb 6;10(2):e0113261. doi: 10.1371/journal.pone.0113261 (PMC4320064; doi:10.1371/journal.pone.0113261)
Supplement: S2 Table — (μ) mean yields in kg ha-1 and (σ2) variance of the upper and lower components of the aggregated (all treatments, n = 434) and treatment-conditional (n = 31) corn yield density distributions. Crop abbreviations: C = Corn, S = Soybean, A = Alfalfa, W = Winter wheat, O = Oat, B = Spring barley, rc = under seeded red clover. (DOCX) [file pone.0113261.s009.docx]

**Supporting Information Table S2.** Parameters of the estimated yield distribution mixture models

|  | $\lambda_{1}$ | $\lambda_{2}$ | $\mu_{1}$ | $\mu_{2}$ | $\sigma_{1}^{2}$ | $\sigma_{2}^{2}$ |
| --- | --- | --- | --- | --- | --- | --- |
| All treatments pooled | 0.796 | 0.204 | 8959 | 11018 | 1745270 | 426280 |
| *Tillage* |  |  |  |  |  |  |
| CCCC | 0.740 | 0.260 | 8507 | 11023 | 1294706 | 540076 |
| CCSS | 0.307 | 0.693 | 7883 | 10167 | 1659123 | 1197380 |
| CCAA | 0.246 | 0.754 | 8016 | 10206 | 1075317 | 1495506 |
| CCSW | 0.232 | 0.768 | 7479 | 9810 | 1656732 | 1660532 |
| CCOB | 0.153 | 0.847 | 7186 | 9703 | 1584807 | 1390996 |
| CCSWrc | 0.196 | 0.804 | 7843 | 10159 | 1462817 | 1402251 |
| CCOrcBrc | 0.096 | 0.904 | 7453 | 9935 | 1755207 | 1343680 |
|  |  |  |  |  |  |  |
| *Reduced Tillage* |  |  |  |  |  |  |
| CCCC | 0.715 | 0.285 | 8203 | 10371 | 1413730 | 535580 |
| CCSS | 0.702 | 0.298 | 8767 | 10685 | 1433856 | 312530 |
| CCAA | 0.557 | 0.443 | 8583 | 10827 | 817408 | 657791 |
| CCSW | 0.200 | 0.800 | 7501 | 9425 | 1359910 | 1440722 |
| CCOB | 0.580 | 0.420 | 8500 | 10448 | 1294091 | 417143 |
| CCSWrc | 0.699 | 0.301 | 8732 | 10594 | 1196755 | 427449 |
| CCOrcBrc | 0.731 | 0.269 | 9118 | 11067 | 1224219 | 424920 |
