# Supplementary material for: Increasing Crop Diversity Mitigates Weather Variations and Improves Yield Stability
Source: PLoS One. 2015 Feb 6;10(2):e0113261. doi: 10.1371/journal.pone.0113261 (PMC4320064; doi:10.1371/journal.pone.0113261)
Supplement: S3 Table — F-statistic without treatment fixed-effects indicates that including a time trend provides a statistically significant improvement in fit over an intercept-only model while adding treatment effects did not; therefore, we use the parsimonious model without treatment effects (note the qualitative results from the analysis in Table 2 do not change under different trend assumptions). The log-linear regression of non-constant yield variance is based on the empirical heteroscedasticity coefficient procedure of [108] and fails to reject the null hypothesis of homoscedasticity. Therefore, we did not include a correction for non-constant variance and the temporally adjusted (i.e. detrended) yields are re-centered to 2012 based on the estimated time trend. (DOCX) [file pone.0113261.s010.docx]

**Supporting Information Table S3.** Estimated temporal pattern in mean and corn yield variance.

| **Student's t-test of trend regression without treatment effects** | | | | | | |
| --- | --- | --- | --- | --- | --- | --- |
|  | Estimate | Robust Std.E | t-value | Pr(>\|t\|) |  |  |
| (Intercept) | 7791.1 | 133.7 | 58.3 | 2.20E-16 | *** |  |
| Trend | 51.2 | 7.0 | 7.3 | 1.16E-12 | *** |  |
|  |  |  |  |  |  |  |
| **Successive F-tests of joint significance from adding treatment effects** | | | | | | |
|  | *df* | Sum of Squared Error | Mean Squared Error | F-value | Pr(>F) |  |
| Trend | 1 | 91169488 | 91169488 | 41.056 | 0.000 | *** |
| Intercepts | 13 | 28207433 | 2169803 | 0.977 | 0.473 |  |
| Slopes | 13 | 9588931 | 737610 | 0.332 | 0.987 |  |
| **Student's t-test of heteroscedasticity coefficient** | | | | | | |
|  | Estimate | Robust Std.E | t-value | Pr(>\|t\|) |  |  |
| (Intercept) | 13.858455 | 18.2478 | 0.7595 | 0.448 |  |  |
| Log(Fitted Values) | -0.054942 | 2.016044 | -0.0273 | 0.9783 |  |  |
