# Supplementary material for: Increasing Crop Diversity Mitigates Weather Variations and Improves Yield Stability
Source: PLoS One. 2015 Feb 6;10(2):e0113261. doi: 10.1371/journal.pone.0113261 (PMC4320064; doi:10.1371/journal.pone.0113261)

**Supporting Information Table S7.** Overall performance index under alternative trend and density estimation assumptions.


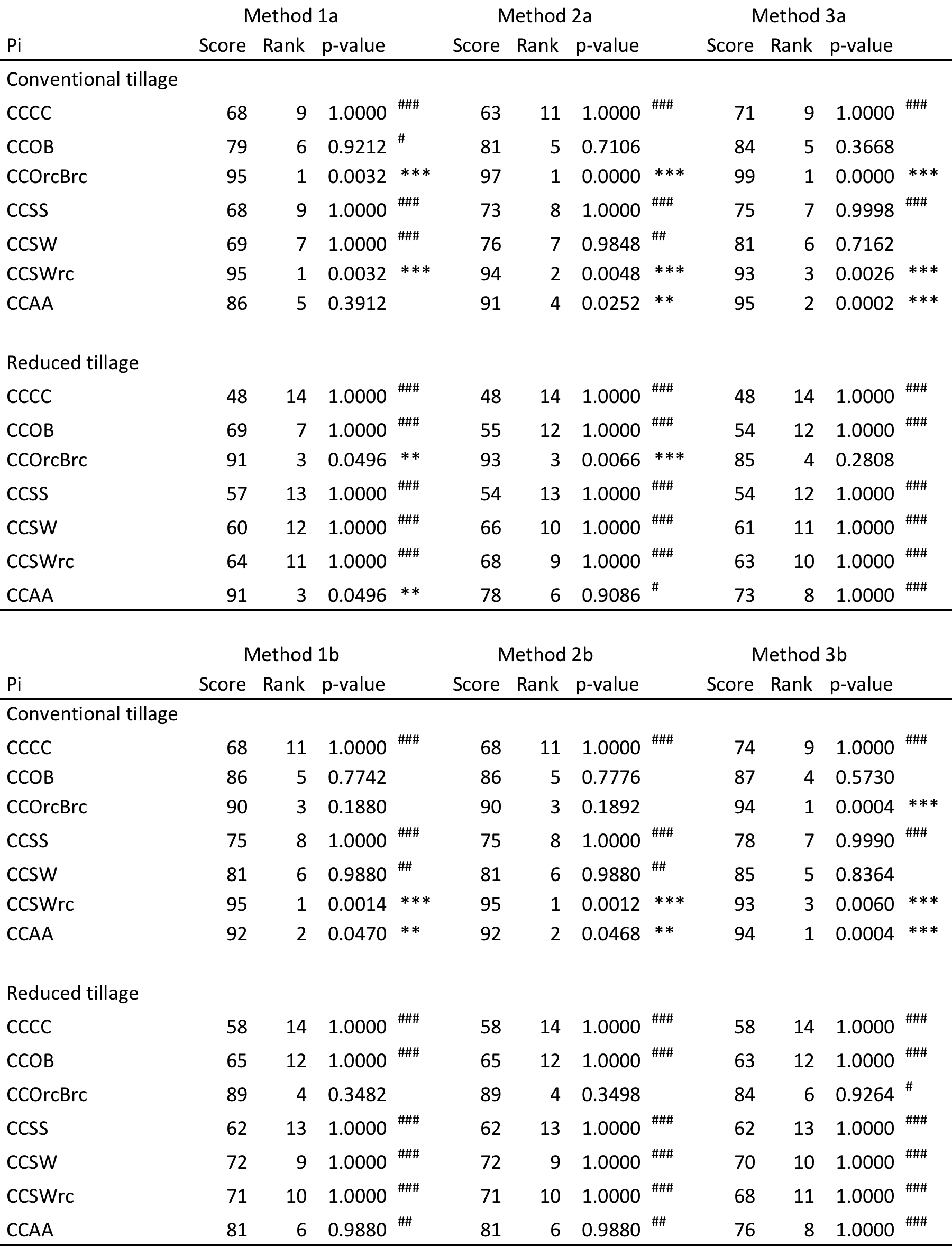

Supplement: S7 Table — Trend (1–3) and density (a-b) estimation methods: (1) linear without treatment effects; (2) linear trend estimation with treatment effects; (3) nonparametric local regression trend; (a) mixture of two Normals; (b) nonparametric kernel density estimate. (DOCX) [file pone.0113261.s014.docx]
